# Chem-map profiles drug binding to chromatin in cells

**DOI:** 10.1038/s41587-022-01636-0

**Published:** 2023-01-23

**Authors:** Zutao Yu, Jochen Spiegel, Larry Melidis, Winnie W. I. Hui, Xiaoyun Zhang, Antanas Radzevičius, Shankar Balasubramanian

**Affiliations:** 1 Yusuf Hamied Department of Chemistry, University of Cambridge, Cambridge, UK; 2 Cancer Research UK Cambridge Institute, University of Cambridge, Cambridge, UK; 3 School of Clinical Medicine, University of Cambridge, Cambridge, UK

**Keywords:** Small molecules, Chemical tools, DNA

## Abstract

Characterizing drug–target engagement is essential to understand how small molecules influence cellular functions. Here we present Chem-map for in situ mapping of small molecules that interact with DNA or chromatin-associated proteins, utilizing small-molecule-directed transposase Tn5 tagmentation. We demonstrate Chem-map for three distinct drug-binding modalities as follows: molecules that target a chromatin protein, a DNA secondary structure or that intercalate in DNA. We map the BET bromodomain protein-binding inhibitor JQ1 and provide interaction maps for DNA G-quadruplex structure-binding molecules PDS and PhenDC3. Moreover, we determine the binding sites of the widely used anticancer drug doxorubicin in human leukemia cells; using the Chem-map of doxorubicin in cells exposed to the histone deacetylase inhibitor tucidinostat reveals the potential clinical advantages of this combination therapy. In situ mapping with Chem-map of small-molecule interactions with DNA and chromatin proteins provides insights that will enhance understanding of genome and chromatin function and therapeutic interventions.

## Abstract (web summary)

How small molecules bind chromatin and DNA is determined by Chem-map.

## Main

Small molecules that interact with cellular DNA formed the basis for early anticancer molecules that became widely deployed in the clinic^1^. A greater understanding of genome structure and function has created new opportunities for intervening with biology and disease states by targeting cellular DNA or the associated proteins with small molecules. It is essential to validate target engagement for molecular probes and therapeutic drugs. Mapping where a small molecule binds chromatin can help explain the downstream genetic or epigenetic response^2^. However, mapping inhibitors to chromatin-associated proteins has proved challenging and is limited to a few high-affinity ligands, including the bromodomain inhibitor JQ1 and the CDK9 inhibitor AT7519 using Chem-seq or related approaches^3–6^. Existing methods involve the binding of small molecules to sheared chromatin followed by enrichment using an affinity tag. Such approaches require high ligand binding affinity and low dissociating rates and suffer from weak signal and high background, together with potential epitope masking upon excessive formaldehyde cross-linking. Furthermore, the requirement for large amounts of input material (typically tens of millions of cells) precludes applications involving low cell numbers or rare epitopes^3^. The detection of binding sites for small molecules that bind directly to cellular DNA has proved to be largely elusive. Some binding sites have been inferred by mapping downstream DNA damage response or break events^7–11^. While direct detection has been achieved for DNA minor groove binding molecules to synthetic DNA oligonucleotides^12,13^ and for the intercalator psoralen UV-crosslinked to DNA in cells^3,14^, a practical challenge for noncovalent small-molecule–DNA interactions is dissociation during washing steps and sample processing^4^. Therefore, many approved, widely used drugs thought to act via a DNA targeting modality have, remarkably, not yet had their cellular molecular targets validated. In situ maps of small-molecule–DNA interactions in intact cells would provide valuable insights into the mode of action of this family of drugs and enhance our ability to exploit the genome as a therapeutic target.

Herein we report a general approach, Chem-map, to establish in situ interaction maps for small molecules that bind to cellular genomic DNA or chromatin-associated proteins. We illustrate the method with three distinct interaction modalities for molecules that either target a chromatin protein, a DNA secondary structure or intercalate DNA.

## Results

### Chem-map of a small-molecule–chromatin protein interaction

**Fig. 1:**
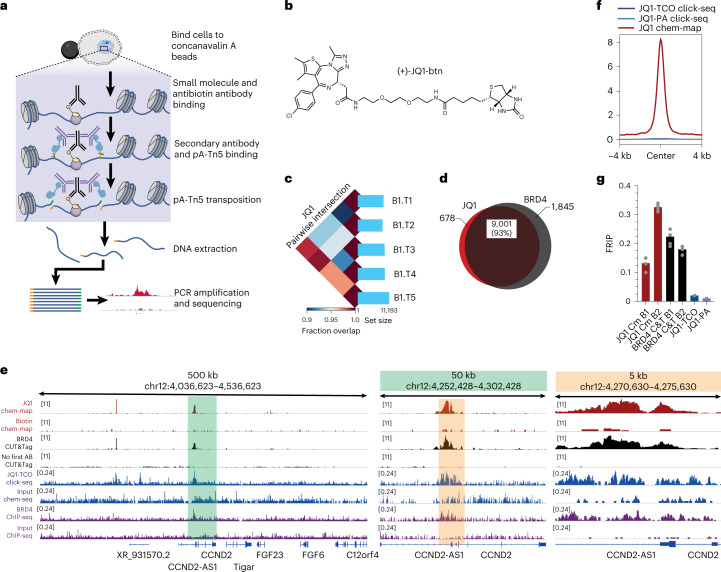
Chem-map reveals genomic binding sites for the BET bromodomain-targeting drug JQ1. **a**, Chem-map workflow—in permeabilized cells, a precomplex of biotinylated small molecules (yellow) and antibiotin antibody (black) bind to the chromatin target (protein or DNA). Then a secondary antibody (purple) tethering of pA–Tn5 transposomes (light blue) was recruited to the drug binding sites. Addition of Mg^2+^ activates the transposomes and integrates adapters (green and orange) in the proximity of the drug-binding sites. After DNA purification, genomic fragments with adapters at both ends are enriched via PCR, which allows the genome-wide identification of drug-binding sites by next-generation sequencing. **b**, Chemical structure of biotinylated JQ1. **c**, Pairwise intersection of enriched peaks across five technical replicates (T1−T5) in a biological replicate (B1) of K562 cells. **d**, Venn diagram illustrating the overlap of high-confidence binding sites of JQ1 (Chem-map, red) and its protein target BRD4 (CUT&Tag, black) in K562 cells. **e**, Genome browser views of JQ1 Chem-map (red), biotin negative control (red) and BRD4 CUT&Tag (black) and its negative control without adding primary antibody (first Ab), compared to published JQ1 Click-Chem-seq (blue) and BRD4 ChIP–seq (purple) data at the *CCND2* gene locus. Green and orange boxes highlight regions of respective close-up views. **f**, FRiP analysis comparing JQ1 Chem-map (Cm, *n* = 5), BRD4 CUT&Tag (C&T, *n* = 5), JQ1 Click-Chem-seq (Click-seq, different JQ1 derivatives JQ1-TCO and JQ1-PA, *n* = 1) and BRD4 ChIP–seq in K562 cells. **g**, Comparison of JQ1 Chem-map and published JQ1 Click-Chem-seq signal averaged at highest-confidence loci detected with BRD4 CUT&Tag in K562 cells. All sequencing data are normalized by sequencing depth.

In the Chem-map approach, a covalent affinity tag is introduced to the small molecule and exploited to recruit a transposase (Tn5) to the binding site, followed by marking the site via proximal transposition events (Fig. 1a). Tn5 recruitment to proteins has been deployed in TAM-ChIP and CUT&Tag^15^. We introduced a preassembled complex, comprising a biotinylated small molecule together with antibiotin primary antibody, directly into permeabilized cells enabling the small molecule to bind the target. An anti-Ig secondary antibody precomplexed with a protein A–Tn5 fusion, loaded with sequencing adapters, is then incubated with the cells. Activation of the transposome with magnesium ions then triggers the insertion of sequencing adapters proximal to the small-molecule-binding sites. Tagmented DNA fragments that mark the small-molecule-binding sites are then extracted, selectively amplified, sequenced and mapped by alignment of sequenced reads to the genome.

**Extended Data Fig. 1:**
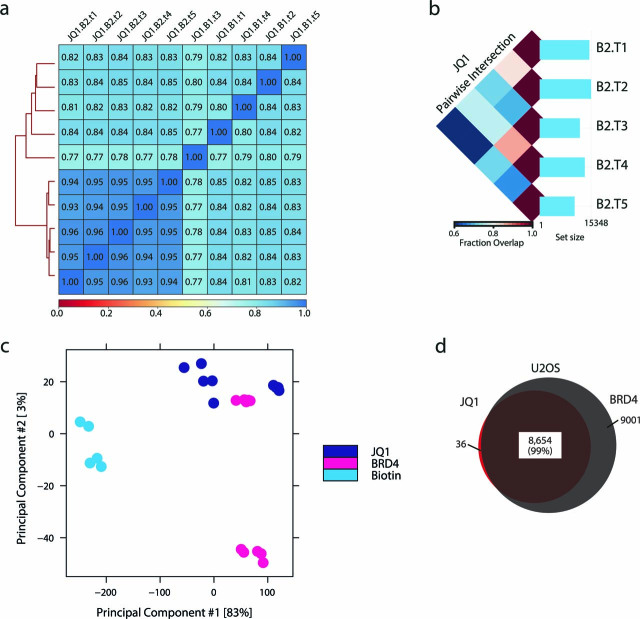
Characterization of Chem-map for JQ1. (**a**) Hierarchical clustering of the Spearman correlation matrix for two biological replicates of JQ1 Chem-map in K562 cells each with 5 technical replicates. (**b**) Pairwise intersection of enriched peaks from JQ1 Chem-map in K562 cells for a 2^nd^ biological replicate comprising five technical replicates. (**c**) Principal components analysis (PCA) comparing JQ1 Chem-map, biotin Chem-map, and BRD4 CUT&Tag in K562 cells. (**d**) Venn diagram illustrating the high-confidence binding sites of JQ1 (Chem-map, red) and its protein target BRD4 (CUT&Tag, black) in U2OS cells.

We validated Chem-map for the BET inhibitor JQ1 in human leukemia K562 cells, as JQ1 interactions have been characterized using Click-Chem-seq in the same cell line^5^. In parallel, we mapped the genome-wide binding sites of the inhibitor’s main target BRD4 using a specific antibody and the CUT&Tag approach^15^. Chem-map experiments were performed with the biotinylated derivative JQ1-btn (Fig. 1b) and biotin to control for background binding of the pA–Tn5 complex. We performed two biological replicates, each with five technical replicates, to evaluate the robustness and technical reproducibility of our approach (Supplementary Table 1). In each experiment, we observed around 10,000 JQ1 binding sites and high reproducibility of Spearman correlation *r*_S_ > 0.77 across replicates (Fig. 1c and Extended Data Fig. 1a,b). Next, we compared high-confidence JQ1 binding sites (Methods) obtained via Chem-map with BRD4 sites obtained via CUT&Tag and found 93% of JQ1 peaks overlap with BRD4 sites (Fig. 1d). Principal components analysis (PCA) confirmed that JQ1 and BRD4 data cluster together and are separated qualitatively from the biotin control (Extended Data Fig. 1c). Thus, Chem-map accurately captures JQ1 binding in cells. Similar observations were made in parallel experiments using the human osteosarcoma epithelial U2OS cells (Extended Data Fig. 1d).

We quantitatively compared the data quality of Chem-map to the published JQ1 Click-Chem-seq data in K562 cells^5^. Chem-map exhibited improved raw data quality yielding ~20-fold higher fraction of reads in peaks (FRiP) compared to Click-Chem-seq and is similar to CUT&Tag (Fig. 1e,f). We also plotted the average read counts for Chem-map and Click-Chem-seq around the highest-confidence BRD4 binding sites obtained by BRD4 CUT&Tag. Chem-map provided ~150-fold higher signal accumulation compared to Click-Chem-seq (maximum mean read count 8.17 counts per million (cpm) and 0.05 cpm, respectively) (Fig. 1g). This improvement in signal quality was observed while using 1 × 10^5^ cells per experiment in Chem-map.

### In situ mapping of DNA G-quadruplex binding

**Fig. 2:**
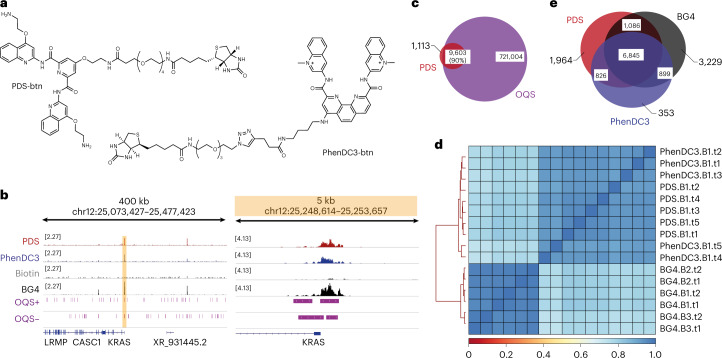
Chem-map reveals genomic binding sites of DNA G-quadruplex binding molecules in human K562 cells. **a**, Chemical structure of biotinylated G4 ligands PDS and PhenDC3. **b**, Genome browser views of Chem-map for PDS (red) and PhenDC3 (blue) compared to CUT&Tag data for the G4 antibody BG4 (black) at the *KRAS* locus. Sites that fold into G4 structures in vitro (OQs) are highlighted in purple for the plus and minus strands. The orange box highlights the regions of a close-up view. **c**, Venn diagrams illustrating the overlap of binding sites for the G4 ligand PDS and OQs. **d**, Hierarchical clustering of the Spearman correlation matrix for PDS Chem-map, PhenDC3 Chem-map and BG4 CUT&Tag. **e**, Venn diagrams illustrating the overlap of binding sites for the G4 ligands PDS and PhenDC3 and BG4.

We next sought to map the binding sites of two structurally distinct DNA G-quadruplex (G4) recognizing molecules, pyridostatin (PDS) and PhenDC3 (Fig. 2a)^16,17^. G4s are four-stranded secondary structures that can form in G-rich DNA sequences. They have been detected in human cells and tissues and mapped in human chromatin using antibody approaches^18,19^. G4s have been implicated in gene regulation, cell fate transitions, and cancers and are under investigation as therapeutic targets for small-molecule drugs^20^. For instance, the G4 ligand PDS has been shown to modulate transcription, cause replication stalling and induce particular patterns of DNA damage^7^. There is a pressing need for a methodology to confirm that such molecules actually bind to G4 targets in situ and to elucidate those binding sites in different biological contexts. This is particularly relevant as the endogenous landscape of cellular G4 that are detectable appears to be only a small fraction of possible G4s in the genome^18^. Attempts to directly map the binding sites of G4 ligands in cells have been unsuccessful. Enrichment sequencing via pulldown of isolated genomic DNA only provided evidence of ligand binding to repetitive telomeric elements^21^. Indirect evidence has arisen from mapping surrogates such as γH2AX sites in the region of strand breaks caused by the binding events of G4 ligand PDS^7^. A likely limitation is small-molecule dissociation from the DNA target by physical separation during washing steps leading to low recovery and poor signal. Indeed, single-molecule imaging studies have shown that ligand binding lifetime to G4s in live cells is in the order of seconds^22^. We reasoned that a transposome-based method (Fig. 1a) might detect dynamic, noncovalent DNA–small-molecule interactions where the lifetime is sufficient for catalytic adapter insertion in situ.

**Extended Data Fig. 2:**
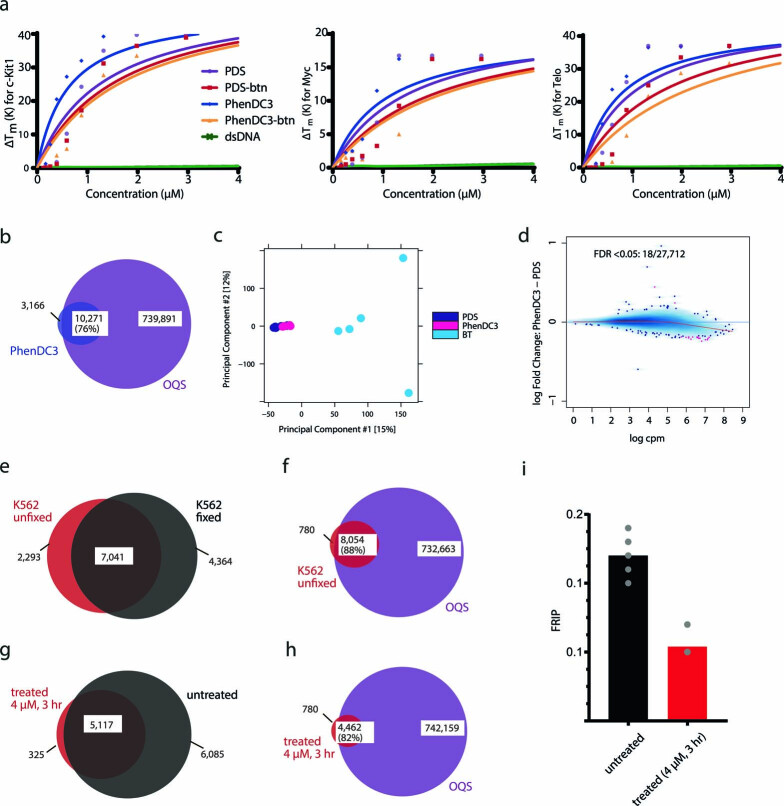
Chem-map data for G4 ligands (PDS and PhenDC). **(a)** FRET-melting assay of G4 ligand binding to G4-forming oligonucleotides (c-Kit, Myc, and Telo) and negative control dsDNA with inducing thermal stabilization (Δ*T*_m_). Mean is represented from two independent experiments (*n* = 2). (**b**) Venn diagrams illustrating the overlap of binding sites for the G4 ligands PhenDC3 and OQS. (**c**) Differential binding analysis for PDS Chem-map and PhenDC3 Chem-map. (**d**) Principal components analysis (PCA) comparing Chem-map experiments with G4 ligands and biotin control in K562 cells. (**e**) Venn diagrams illustrating the overlap of PDS Chem-map high-confidence peaks from lightly fixed and unfixed K562 cells (present in 3/5 technical replicates) and the respective overlap with OQS (**f**). (**g**) Venn diagrams illustrating the overlap of PDS Chem-map high-confidence peaks from K562 cells (present in 3/5 technical replicates) dosed with unmodified PDS (4 μM for 3 h) and overlap of those peaks with OQS (**h**). (**i**) FRiP analysis comparing untreated and treated (unmodified PDS, 4 μM for 3 h) PDS Chem-map in K562 cells (*n* = 5).

Biotinylated G4 ligands PDS-btn and PhenDC3-btn were synthesized with a flexible, long PEG4 linker to minimize steric hindrance to recognition of the antibody binding and subsequent targeted DNA tagmentation (Fig. 2a). A fluorescence resonance energy transfer (FRET)-melting assay was employed to validate the binding of the tagged G4 ligands to different G4s (Extended Data Fig. 2a and Supplementary Tables 2 and 3). We employed both PDS-btn and PhenDC3-btn in our Chem-map protocol in K562 cells, employing biotin as a negative control and performing two biological replicates each with five technical replicates. For both G4 ligands, we obtained high-quality maps revealing about 10,000 high-confidence binding sites for each of the two G4 ligands (Fig. 2b). It was gratifying to observe that PDS and PhenDC3 binding sites showed considerable overlap (90% for PDS and 76% for PhenDC3, respectively) with where G4s can potentially form experimentally observed G-quadruplexes (OQs) based on G4-sensitive sequencing (G4-seq) of purified human genomic DNA (Fig. 2c and Extended Data Fig. 2b)^23^. We also compared the small-molecule-binding sites to maps of endogenous G4s detected in chromatin using the G4 antibody BG4 (Fig. 2d)^24^. We observed a strong overlap of high-confidence binding sites (Fig. 2e) and signal correlation when comparing BG4 antibody and G4 ligands with Spearman correlations (*r*_s_) of 0.74 and 0.87 for PDS and PhenDC3, respectively (Fig. 2d). Similarly, differential binding analysis revealed that PDS and PhenDC3 bind primarily to the same cellular G4 sites (Extended Data Fig. 2c). Notably, PCA analysis confirmed clear qualitative separation of the biotinylated probes from the biotin control experiments confirming that signal enrichment is caused by the small molecule rather than nonspecific interactions of Tn5 (Extended Data Fig. 2d). To rule out potential fixation artifacts^25^, we repeated PDS Chem-map in unfixed K562 cells observing an overall consistent peak distribution (~10,000 high-confidence peaks, 88% in the OQs and 75% overlap with binding sites in gently fixed K562 cells) (Extended Data Fig. 2e,f). Furthermore, to confirm that Chem-map reflects small-molecule-binding sites in live cells, we treated K562 cells with unmodified PDS (4 µM for 3 h) before PDS Chem-map (Extended Data Fig. 2g−i). We observed a drop in recovered DNA material and a considerable reduction in peak numbers (~6,000 sites (60%) lost compared to untreated).

These data obtained within human cells confirm that PDS, PhenDC3 and BG4 are specific to the G4 family of DNA structures and capable of binding a variety of G4 sequences and structural sub-types, consistent with biophysical studies^18,23^. Notably, the small-molecule Chem-map data provide vital cross-validation of cellular G4 sites previously detected using the BG4 antibody.

### Chem-map reveals landscape of doxorubicin–DNA interactions

We evaluated the interactions of the clinically approved drug doxorubicin, which is thought to act by targeting DNA but has not yet been directly mapped to genomic DNA in cells. Doxorubicin belongs to the anthracycline class of antitumor antibiotics. Anthracyclines are generally considered to be DNA intercalators that can inhibit the action of topoisomerase II and can form reactive hydroxyl radicals proximal to DNA, leading to DNA damage and cellular cytotoxicity^1^. Around 1 million cancer patients annually receive treatment with doxorubicin or its variants. However, despite much research and clinical use over five decades, its molecular mode of action is, somewhat surprisingly, still not well understood^26^.

**Fig. 3:**
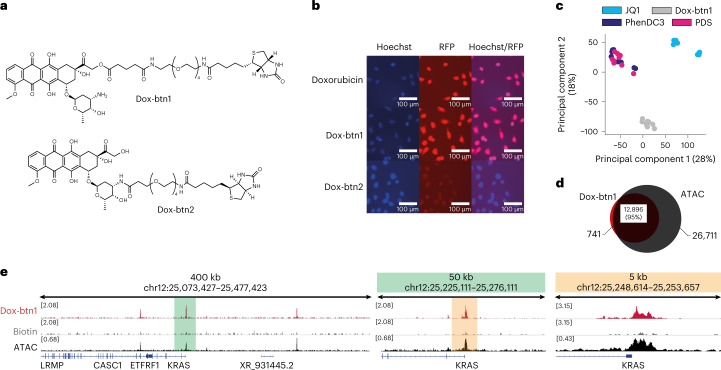
Chem-map reveals an open chromatin binding preference for doxorubicin. **a**, Chemical structure of biotinylated derivatives of doxorubicin. **b**, Microscopy analysis of U2OS cells visualizing nuclear enrichment of doxorubicin derivatives. Live U2OS cells were treated with doxorubicin and its derivatives for 6 h. Nuclei were stained with Hoechst 33342. BFB was used for visualization of nuclei (blue) and RFB was used for visualization of doxorubicin and its derivatives (red). Scale bar, 100 μm. Experiments were repeated independently three times. **c**, PCA analysis showing the distinct binding profiles of small molecules that have different protein and DNA targets in K562 cells. **d**, Venn diagram showing the overlap of doxorubicin binding sites with open chromatin mapped by ATAC-seq. **e**, Genome browser views of doxorubicin Chem-map binding sites (red) compared to a biotin control (gray) and ATAC-seq (black). BFB, blue-light filter cube; RFB, red-light filter cube.

**Extended Data Fig. 3:**
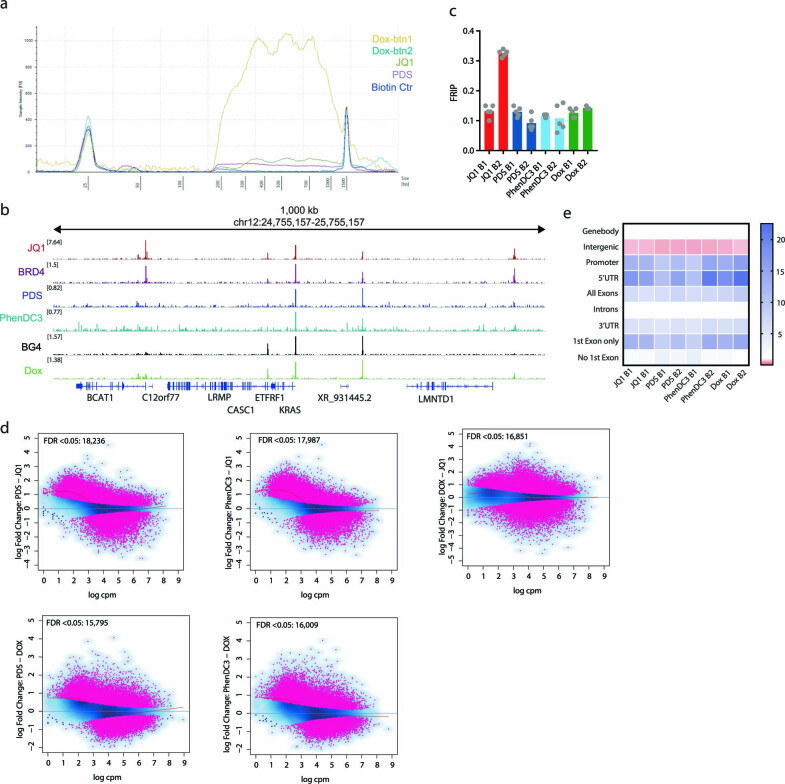
Characterization of Chem-map datasets with different small molecules. (**a**) Tapestation analysis comparing relative DNA recovery after PCR for Dox-btn1, Dox-btn2, JQ1-btn, PDS-btn, PhenDC3-btn, and biotin control. (**b**) Gene browser views of peak profiles among JQ1 Chem-map, BRD4 CUT&Tag, PDS Chem-map, PhenDC3 Chem-map, BG4 CUT&Tag, and doxorubicin Chem-map. (**c**) FRiP analysis of Chem-map with different small molecules in K562 cells. (**d**) Differential binding analysis (FDR < 0.05) for Chem-map comparing binding sites for different small molecules in K562 cells. Red dots represent sites where the binding is significantly different for two molecules (considering two biological replicates each with five technical replicates). (**e**) Enrichment over random (n = 1,000 permutations) of JQ1, PDS, PhenDC3 and Doxorubicin at genomic features from the reference human annotation GENECODE v.28. UTR untranslated region; Promoter defined as 1 kb upstream transcription start sites. Peak distribution of Chem-map experiment with JQ1, PDS, PhenDC3, and doxorubicin, across different genomic features.

For Chem-map, it was necessary to design an appropriately tagged derivative of doxorubicin. We evaluated two points of conjugation to doxorubicin at 14-OH and 3′-NH_2_ resulting in biotinylated derivatives Dox-btn1 and Dox-btn2, respectively (Fig. 3a). Next, we examined the cellular distribution in U2OS cells using fluorescence microscopy utilizing the intrinsic fluorescence of doxorubicin (Fig. 3b). Notably, doxorubicin and Dox-btn1 were predominantly accumulated in the nuclei, whereas Dox-btn2 was mainly located in the cytoplasm, in agreement with a previous study^27^. Negligible amounts of DNA were recovered using Chem-map with Dox-btn2 in K562 cells, whereas Dox-btn1 recovered substantial amounts of DNA (Extended Data Fig. 3a), consistent with its nuclear localization.

Biophysical experiments show that doxorubicin intercalates double-stranded DNA preferentially at a GC base pair^28^, which does not in itself inform genomic binding sites. Chem-map data from two biological and five technical replicates in K562 cells revealed 14,000 high-confidence Dox-btn1 binding sites (Fig. 3e and Extended Data Fig. 3b), with an excellent signal-to-noise ratio (Extended Data Fig. 3c). We compared Dox-btn1 binding sites to the binding sites of JQ1, PDS and PhenDC3, each measured by Chem-map. Both PCA (Fig. 3c) and differential binding analyses (Extended Data Fig. 3d) revealed distinct binding profiles for the three types of ligands, caused by the different target binding modalities for the respective probes. The 14,000 doxorubicin peaks were predominantly (95%) in open chromatin regions, as defined by ATAC-seq^29^ (Fig. 3d,e). Given that Tn5-based approaches can map features in both euchromatin and heterochromatin^15^, enrichment of Dox-btn1 at open chromatin is unlikely to be an artifact of Tn5 accessibility. Notably, we found Dox-btn1 binding sites particularly enriched at promoters and 5′-untranslated regions (Extended Data Fig. 3e), which helps explain why doxorubicin preferentially induces DNA double-strand breaks near promoters^30^.

### HDACi sensitizes cancer to doxorubicin by augmenting binding

**Fig. 4:**
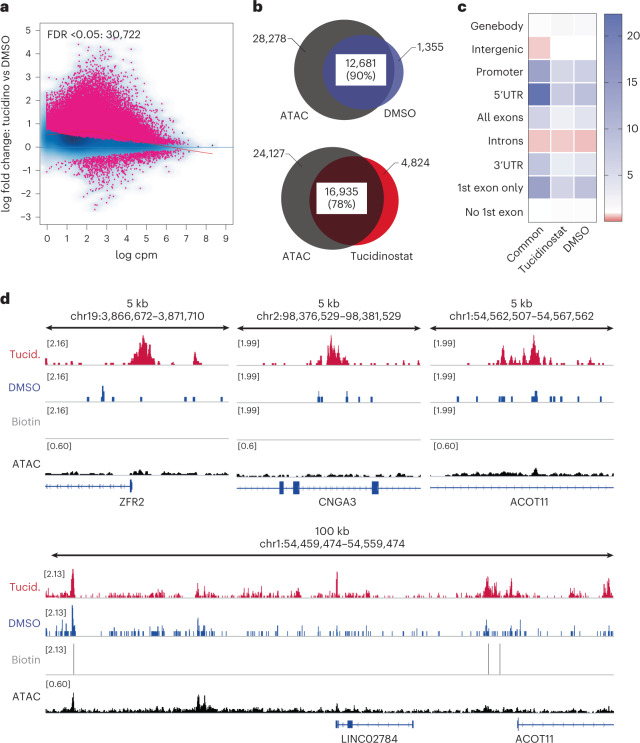
Chem-map reveals response to drug combinations. **a**, Differential binding analysis showing differences in Dox-btn1 Chem-map peaks for tucidinostat-treated (1 µM, 72 h) and vehicle-treated (0.1% DMSO, 72 h) K562 cells. Red dots represent sites where the binding is substantially different (FDR < 0.05) for two treatments (considering three biological replicates each with five technical replicates). A positive fold change indicates an increase in Dox-btn1 binding. **b**, Venn diagram illustrating the overlap of high-confidence Dox-btn1 binding sites with ATAC-seq in vehicle-treated (blue) and tucidinostat-treated (red) K562 cells. **c**, Enrichment over random (*n* = 1,000 permutations) of Dox-btn1 Chem-map peaks at genomic features from the reference human annotation GENECODE v.28. Up/down, substantially up- or down-regulated peaks in tucidinostat-treated K562 cells. Promoter defined as 1 kb upstream transcription start sites. **d**, Genome browser views displaying the difference in Dox-btn1 binding for tucidinostat-treated (red) or vehicle-treated (blue) cells in K562 cells compared to a biotin control (gray) and ATAC-seq (black). UTR, untranslated region.

**Extended Data Fig. 4:**
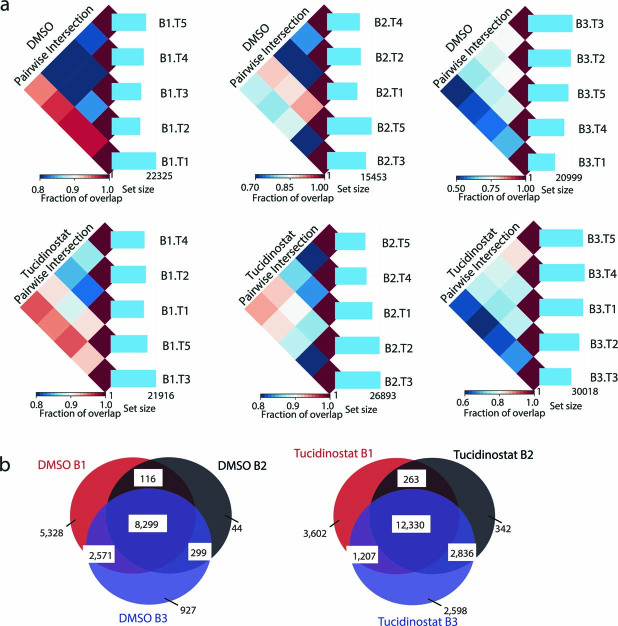
Characterization of Dox-btn1 Chem-map for tucidinostat. (**a**) Pairwise intersection of enriched peaks from Dox-btn1 Chem-map for DMSO vehicle control- and tucidinostat-treated K562 cells. (**b**) Venn diagram comparing high-confidence binding sites for three biological replicates of Dox-btn1 Chem-map in vehicle control- and tucidinostat-treated K562 cells.

The dynamic nature of chromatin status makes it difficult to predict and control the response to chromatin-targeted drugs in cells^4^. Chem-map enables the measurement of changes in drug binding profiles to help understand the mode of action and optimize their deployment. To exemplify this, we applied Chem-map to measure how epigenetic modulators can enhance DNA–drug interaction to create drug synergy. Specifically, we investigated how doxorubicin binding in cells changes upon treatment with a histone deacetylases inhibitor (HDACi). Histone deacetylases (HDAC) are key chromatin modifiers and commonly dysregulated in cancers, making them promising therapeutic targets for cancer. Preclinical and clinical studies show that HDAC inhibition can sensitize the response of cancer to doxorubicin treatment^31,32^. We chose to use tucidinostat (chidamide), a selective inhibitor of class I HDAC1−HDAC3 and class IIb HDAC10, which has been clinically approved for peripheral T-cell lymphoma and adult T-cell leukemia-lymphoma^33,34^. Preclinical and clinical trials of tucidinostat in combination with doxorubicin are ongoing^35^ (NCT04231448). We treated K562 leukemia cells with tucidinostat (1 μM) for 72 h, followed by Chem-map with Dox-btn1. From three biological replicates, each with five technical replicates, we observed excellent reproducibility and clear separation of tucidinostat treatment and vehicle control (Extended Data Fig. 4). Tucidinostat treatment resulted in a substantial shift in Dox-btn1 binding events (30,722 substantially changing sites), mainly comprising stronger binding events and new binding sites, as judged by differential binding analysis (Fig. 4a,d). More doxorubicin binding events were detected at sites that had originally exhibited closed chromatin in the absence of tucidinostat treatment (22% in tucidinostat compared with 10% in vehicle group), consistent with considerable chromatin remodeling (Fig. 4b). In addition, new peaks observed following tucidinostat treatment were mainly located at promoters and 5′-untranslated regions (Fig. 4c,d). Thus, HDAC inhibitors likely sensitize cancer cells via expanding existing drug-binding sites and establishing new interaction sites to enhance the overall volume of drug–target interactions. These molecular data explain clinically relevant drug synergistic effects^31,32^.

## Discussion

There is a considerable demand for a robust and general approach to map the interaction between small molecules and chromatin in situ. Small molecules can target chromatin in ways that interfere with vital processes that include DNA replication, transcription, DNA repair, DNA and histone modifications, and epigenetic reprogramming, which has also led to therapeutic drugs. In this study, we show that Chem-map is a robust approach for mapping the interactions of small molecules with genomic DNA and chromatin proteins in cells. Chem-map provides unprecedented signal quality and has generated insights into the genomic targeting of probe molecules and also a clinically relevant drug, which was previously not attainable. Therefore, we expect that Chem-map can be adapted to a wide range of chromatin-interacting molecules and studies involving low cell numbers and rare epitopes. Nonetheless, probe concentrations will have to be titrated depending on the abundance of binding sites and strength of the interaction (also see Supplementary Technical Discussion). For instance, we observed substantially stronger enrichment for the intercalator doxorubicin compared to the protein binder JQ1 or the DNA G4 binding ligands. While gentle formaldehyde fixation is commonly used to avoid decomposition or clumping of permeabilized cells during the protocol^36^ and can be omitted in certain cases (see for example PDS Chem-map), fixation becomes relevant when small-molecule probes directly compete for chromatin binding domains of the target proteins. In these cases, formaldehyde concentrations and cross-linking times should be titrated to allow for efficient recovery, while still limiting fixation artifacts such as epitope masking^25^. In addition, the attachment of the biotin linker will have to be performed such that small-molecule target binding is not impaired and will either require an intricate understanding of the underlying structure–activity relationship or the synthesis of various derivatives (Fig. 3a). Ideally, this process should be guided by some form of functional (Fig. 3b) or biophysical validation of the tagged molecule (Extended Data Fig. 2). Notably, the exploration of suitable linker positions for a variety of small-molecule probes and drugs has considerably increased due to their application in other therapeutic platforms such as antibody-drug conjugates^37^ or proteolysis targeting chimeras^38^.

In addition to the direct mapping of native binding sites, we have shown that Chem-map is able to capture the dynamics of drug binding under changing cellular conditions. We provide a molecular explanation as to how chromatin modulation by epigenetic drugs can enhance the targeting of genomic DNA and sensitize cancer cells to DNA-damaging agents. This is in line with previous observations that cotreatment of a cisplatin derivative with the HDAC inhibitor SAHA or the DNA methyl transferase inhibitor azacytidine activate DNA damage response pathways and increase DNA lesions^39^.

Chem-map will complement other genomics techniques to enable mechanistic studies and further development of genome- and epigenome-targeting drugs.

## Methods

### Cell culture and compound treatment

Mycoplasma-free human chronic myelogenous leukemia K562 cells (RRID:CVCL 0004) were purchased from ATCC and cultured in RPMI 1640 (Gibco, 21875034) supplemented with 10% heat-inactivated FBS (Gibco, A3840401). Human bone osteosarcoma epithelial U2OS cells (RRID:CVCL_0042) derived from a moderately differentiated sarcoma were obtained from ATCC and cultured in DMEM (Gibco, 41966029) supplemented with 10% FBS. Both cell lines were grown in accordance with ENCODE cell culture protocols and tested for mycoplasma contamination and identity confirmed by STR typing.

HDAC inhibitor tucidinostat (also named as Chidamide/HBI-8000/CS-055; Sellect, S8567) was prepared at 10 mM as stock solution in DMSO. Cells were treated with tucidinostat dissolved to 1 μM final concentration or an equal concentration of vehicle (0.1% DMSO) for 72 h.

### FRET melting assay

A total of 400 nM fluorescein (FAM)-5-carboxytetramethylrhodamine dual-labeled oligonucleotides (Biomers; see Supplementary Table 1) was annealed in FRET assay buffer (60 mM potassium cacodylate, pH = 7.4) at 95 °C for 5 min, followed by gradually cooling down to 20 °C. A series of probe concentrations were prepared in an 8-well strip tube as follows: 150 µl of 6 µM ligand in assay buffer was prepared as the initial concentration. Subsequent serial dilutions were made by adding 100 µl of probe solutions to 50 µl of assay buffer, resulting in 12 concentrations, including a control (1% DMSO). A total of 25 µl per solution was transferred to a 96-well plate, followed by adding 25 µl of annealed oligonucleotide solutions to each well. The plate was then sealed with an adhesive transparent cover and shaken gently for 10 min at room temperature. Measurements of restoring FAM signal were recorded on a real-time PCR detection system (Bio-Rad, CFX96) employing a temperature gradient from 25 °C to 95 °C at 0.5 °C min^−1^. Melting temperatures (*T*_m_) were determined by the first derivative maxima of relative fluorescence unit value against time, and ∆*T*_m_ was calculated by baseline correction of melting temperatures subtracting control group. A one-site binding model in GraphPad Prism 8 was used to fit FRET *T*_m_ curves. Mean was calculated from two replicates.

### Cell imaging

U2OS cells were plated in a 12-well plate in DMEM medium supplemented with 10% FBS. After 18 h incubation, cells were treated for 6 h with doxorubicin (1 µM), Dox-btn1 (1 µM), Dox-btn2 (1 µM) and 0.1% DMSO (control), respectively, in fresh medium. Then cells were washed twice with PBS and lightly fixed with 0.1% formaldehyde for 2 min. Cells were then incubated with Hoechst 33,342 for 10 min at room temperature to label the cell nuclei. Imaging was captured in EVOS M5000 microscope (Thermo Fisher Scientific). Blue-light filter cube with wavelength of Exc 357/44 nm and Emi 447/60 nm was used for visualization of nuclei and red-light filter cube with wavelength of Exc 531/40 nm and Emi 593/40 nm was used for visualization of doxorubicin and its derivatives.

### Chem-map protocol

#### Cell preparation

U2OS cells were detached using Accutase (Stem Cell Technologies, 07920) and quenched using complete culture media. U2OS and K562 cells were collected by centrifugation and fixed in 0.1% formaldehyde (Thermo Fisher Scientific, 28906) in PBS for 2 min at room temperature, followed by quenching with glycine (Sigma-Aldrich, 50046) to a final concentration of 75 mM. Fixed cells were collected by centrifugation at 600*g* for 4 min, followed by resuspension in cold wash buffer (20 mM HEPES, pH 7.5, 150 mM NaCl and 0.5 mM spermidine (Sigma-Aldrich, S0266)) in nuclease-free water supplemented with a complete protease inhibitor, EDTA-free (Sigma-Aldrich, 11873580001). (Note: For Chem-map of G4 ligands, NaCl was replaced by equivalent concentrations of KCl in all buffers to maintain G4 stability. For optimal results, the concentration of cells needs to be adjusted based on target abundance and relative affinity of the probes. We employed cells at a concentration of 1,500 cells per μl (JQ1-btn) and 6,000 cells per μl (G4 ligands and biotinylated doxorubicin)).

#### Probe-1st Ab complex assembly

Compound stock solution in DMSO was diluted to 10 µM in antibody buffer (2 mM EDTA, 0.1% BSA (Sigma-Aldrich, A8577) and 0.05% digitonin (EMD Millipore, 300410) in wash buffer). For 5 samples, 20 μl probe solution (10 µM) and 16.7 μl antibiotin (D5A7) Rabbit mAb (Cell Signaling Technology, 5597; concentration is ~10 µM) was added to 200 μl antibody buffer and incubated on ice for 1 h to perform a complex at high concentration (probe in excess 1.2:1 to avoid nonspecific antibody binding). Next, 300 μl antibody buffer was added to the probe-1st Ab complexes solution. The final concentration of small molecule is 0.4 µM. (Note: For Dox-btn1, final concentration is diluted to 0.2 µM. For antibiotin antibody, dilution is 30×).

#### Bead capture

For five samples, 50 μl (for JQ1-btn) or 75 µl (for G4 ligands and biotinylated doxorubicin) concanavalin A beads (Bangs Labs, BP531) were washed twice in 1 ml binding buffer (20 mM HEPES, pH 7.5, 10 mM KCl, 1 mM CaCl_2_ and 1 mM MnCl_2_ in nuclease-free water) and resuspended in 75 µl binding buffer. A total of 100 µl of cell suspension was incubated with 10 µl prewashed concanavalin A beads at 25 °C for 10 min at 600 rpm. Beads-bound cells were gently washed twice with wash buffer before resuspending in 100 µl probe-1st Ab precomplex solution and incubating at 4 °C overnight at 600 rpm.

#### 2nd Ab–Tn5 transposome complex assembly

pA–Tn5 assembled with DNA adapters were prepared as described in ref. ^24^. For 5 samples, 2.5 μl 2nd Ab (Antibodies-Online, ABIN101961, ~8 µM) and 5 μl pA–Tn5 transposome (pA–Tn5 concentration is 2 µM) were added to 200 μl Dig-300 buffer (20 mM HEPES pH 7.5, 300 mM NaCl, 0.5 mM spermidine and 0.01% digitonin in nuclease-free water supplemented with complete protease inhibitor, EDTA-free) and incubated on ice for 1 h (2nd Ab and pA–Tn5 at a ratio of 2:1). A total of 300 μl antibody buffer was added to the 2nd–Tn5 complex solution.

#### Tagmentation

Cells were washed three times with 500 µl Dig-wash buffer (0.05% digitonin in wash buffer) and resuspended in 100 µl 2nd Ab–Tn5 transposome complex solution and incubated at 25 °C for 1 h at 600 rpm. Cells were then washed three times in 500 µl Dig-300 buffer before incubation in 300 µl tagmentation buffer (10 mM MgCl_2_ in Dig-300 buffer) at 37 °C for 1 h at 600 rpm.

#### DNA extraction

After tagmentation, cells were washed twice with 500 µl TAPS wash buffer (10 mM (tris(hydroxymethyl) methylamino) propanesulfonic acid (TAPS; Alfa Aesar, J63268.AE), 0.2 mM EDTA in nuclease-free water). One hundred fifty microliters of extraction buffer (0.5 mg ml^−1^ proteinase K (Thermo Fisher Scientific, EO0491), 0.5% SDS (Sigma-Aldrich, L4509) in 10 mM Tris–HCl, pH 8.0) were added, vortexed and incubated at 55 °C for 1 h at 800 rpm. Next, 150 µl phenol–chloroform–isoamyl alcohol (Invitrogen, 15593049) was added and mixed. The mixture was transferred to MaXtract High-Density phase-lock tubes (QIAGEN, 129046) and centrifuged at room temperature for 3 min at 16,000*g*. A total of 150 µl chloroform was added to the top aqueous phase, mixed by inverting the phase-lock tubes for 10 times, and centrifuged 16,000*g* at room temperature for 3 min. The top aqueous layer was transferred to a 1.5 ml DNA Lo-bind tube (Eppendorf, 022431021). A total of 6 µl 5 M NaCl and 375 µl cold ethanol were added, mixed and incubated at −20 °C overnight. Samples were centrifuged at 21,130*g* at 4 °C for 30 min. The supernatant was carefully poured off and the DNA pellet rinsed with 1 ml cold 100% ethanol followed by centrifugation at 21,130*g* at 4 °C for 2 min. After pouring off the wash and draining the residual liquid with paper towel, the pellet was left to air dry. Finally, the pellet was resuspended in 25 µl elution buffer 1 (10 mM Tris–HCl, pH 8, 1 mM EDTA, 1/400 RNAse A (Thermo Fisher Scientific, EN0531) in nuclease-free water) by vortexing and incubating at 37 °C for 10 min at 800 rpm.

#### Library preparation

In a 0.2 ml PCR tube, 25 µl NEBNext Ultra II Q5 2x PCR master mix (NEB, M0544), 2 µl of 10 µM uniquely barcoded v2 Ad1.x primer^40^, 2 µl of 10 µM uniquely barcoded v2 Ad2.x primer^40^ and 21 µl tagmented DNA were added and subjected to PCR (72 °C for 5 min, 98 °C for 30 s, followed by 10 cycles of 98 °C for 10 s and 63 °C for 10 s and one cycle of 72 °C for 1 min). Libraries were purified using 1.3× ratio (65 µl) Ampure XP beads (Beckman Coulter, A63882). After 10-min incubation at room temperature, bead-bound DNA was washed twice with 80% ethanol and libraries were eluted with 25 µl 10 mM Tris–HCl for 5 min at room temperature. (Note: PCR program condition for Dox-btn1 is 72 °C for 5 min, 98 °C for 2 min, followed by 10 cycles of 98 °C for 10 s and 63 °C for 10 s, and one cycle of 72 °C for 1 min, to maximally disrupt doxorubicin–DNA interaction.)

#### Library sequencing

Library size and concentration were measured using a TapeStation HSD1000 ScreenTape (Agilent, 5067–5584). Libraries were balanced and pooled for size selection using Ampure XP beads. 0.4× ratio of Ampure XP beads was added to pooled libraries and the supernatant transferred to a new tube after 15 min at room temperature. 1.3× ratio of Ampure XP beads was then added to the supernatant and incubated for 15 min at room temperature. Beads were washed twice with 80% ethanol and libraries eluted in 40 µl 10 mM Tris–HCl. Libraries were sequenced on a NextSeq 500 sequencer (Illumina) with a paired-end format of 36 bp × 2 using the High Output kit (Illumina, FC-404-2005).

### CUT&Tag method

pA–Tn5 assembled with DNA adapters were prepared in the lab as described in ref. ^24^. BRD4 CUT&Tag was performed as described before^36^. Briefly, cells were incubated with activated concanavalin A-coated magnetic beads (Bangs Labs, BP531). The bead-bound cells were permeabilized and incubated with anti-BRD4 (E2A7X) rabbit antibody (Cell Signalling Technology, 13440) in antibody buffer at a ratio of 1:50, followed by Guinea Pig anti-rabbit antibody (Antibodies-Online, ABIN101961) in Dig-wash buffer at a ratio of 1:100. Diluted pA–Tn5 adapter complex (1:250 ratio) was then added in Dig-300 buffer followed by the tagmentation reaction in tagmentation buffer. Extracted DNA fragments were used for library preparation and Illumina sequencing.

### Sequencing data processing

#### Data demultiplexing and deduplication

Illumina sequencing paired-end output files were demultiplexed using demuxIllumina version 3.0.9 using the flags; -c -d -i -e -t 1 -r 0.01 -R -l 9. The resulting fq.gz files underwent sequencing quality control using FastQC version 0.11.8, and their summary was visualized by MultiQC version 1.11. Bases with a quality score below 20 were trimmed from both reads using cutadapt (cutadapt -q 20). Fastq files were aligned to the combined hg38 and *Escherichia coli* (*E. coli*) genomes using bwa version 0.7.17-r1188 with only reads in the whitelist regions of hg38 continuing the process pipeline. Duplicates were removed using Picard version 2.20.3 (Picard MarkDuplicates). Peaks were called using Seacr version 1.3 without input control reporting the top 1% by AUC regions, using both the relaxed and stringent criteria. BigWig files were created on the demultiplexed BAM file, normalized at cpm using deepTools version 2.0.

#### Consensus regions and reference comparisons

To address the ambiguity of peak calling, multiple approaches have been used to assess the peak number and overlaps between experiments. Namely, further thresholds have been applied on the Seacr output, creating .bed files containing peaks of minimum ‘total signal’ of threshold of 5, 8 and 10. Taking advantage of the five technical replicates one can also access the reproducibility of a called region. For each threshold, the overlap across the five technical replicates is calculated with intervene tools (Venn upset and pairwise) and a series of .bed files containing at least one (union of all technical replicates) to five (common among all technical replicates) are created using multiIntersectBed with -wa wb flags of bedtools version 2.30.0. Intersection between biological replicates can follow the same pipeline. This classification of peaks allows their quantification and ranking according to the normalized (cpm) signal strength as well as the reproducibility of the peak, allowing weaker but highly reproducible regions to be identified. All correlation analysis among a biological experiment was done on the consensus peaks, and correlation analysis between biological experiments and between different molecules on the union of the corresponding consensus sets, using deepTools^41^. Differential binding analysis follows DiffBind version 3.15 in R package pipeline version 4.2.2, using the deduplicated BAM files and consensus peaks, to create PCA, MA plots as well as calculate FRiP^42,43^. High-confidence peak regions, unless otherwise stated, are considered those regions at the top 1% by AUC, with minimum total signal 5 (min5) and present in three of five replicates (multi3). The highest-confidence *BRD4* binding sites in K562 cells were defined as peaks present across all technical and biological replicates in CUT&Tag (7,772 peaks).

### Statistical analysis

Data are presented as mean ± s.d. The sample sizes (*n*) in the figure legends indicate the number of replicates in each experiment and are provided in the corresponding figure legends. The peak or gene size (*N*) in the heat maps indicates the number of peaks or genes included. Statistical analysis in Fig. 1 and Extended Data Figs. 2–3 was performed by unpaired Student’s *t* tests, and the *P* values were denoted in each figure.

### Reporting summary

Further information on research design is available in the Nature Portfolio Reporting Summary linked to this article.

## Online content

Any methods, additional references, Nature Portfolio reporting summaries, source data, extended data, supplementary information, acknowledgements, peer review information; details of author contributions and competing interests; and statements of data and code availability are available at 10.1038/s41587-022-01636-0.

## Supplementary information

Supplementary Information Supplementary Figs. 1–11, Supplementary Technical Discussion, and Supplementary Tables 1–3.

Reporting Summary

## Data Availability

Sequencing data generated in this study have been submitted to the NCBI Gene Expression Omnibus (GEO; https://www.ncbi.nlm.nih.gov/geo/) under accession number GSE209713 (ref. ^44^). The following previously published datasets were used: hg38 (https://www.ensembl.org/Homo_sapiens/Info/Index), JQ1 Click-Chem-seq (GSE88751)^5^, ATAC-seq (GSE162299)^29^, BG4 CUT&Tag (GSE181373)^24^ and OQs (GSE110582)^23^.

## Code availability

All bioinformatics scripts are available on https://github.com/sblab-bioinformatics/Chem-map ref. ^45^.
