# Supplementary material for: Chem-map profiles drug binding to chromatin in cells
Source: Nat Biotechnol. 2023 Jan 23;41(9):1265–71. doi: 10.1038/s41587-022-01636-0 (PMC10497411; doi:10.1038/s41587-022-01636-0)
Supplement: Supplementary file 1 — Supplementary Figs. 1–11, Supplementary Technical Discussion, and Supplementary Tables 1–3. [file 41587_2022_1636_MOESM1_ESM.pdf]

nature biotechnology

Article https://doi.org/10.1038/s41587-022-01636-0

Chem-map profiles drug binding to chromatin in cells

In the format provided by the authors and unedited

Supplementary information

Chemical synthesis 2
Synthesis of JQ1-btn 3
Synthesis of PDS-btn 4
Synthesis of PhenDC3-btn 5
Synthesis of Dox-btn1 6
Synthesis of Dox-btn2 9
Supplementary Technical Discussion: 11
Supplementary Figure 1. NMR data of JQ1-btn. 13
Supplementary Figure 2. NMR and HRMS data of JQ1-btn. 14
Supplementary Figure 3. NMR data of PDS-btn. 15
Supplementary Figure 4. NMR and HRMS data of PDS-btn. 16
Supplementary Figure 5. NMR data of PhenDC3-yne. 17
Supplementary Figure 6. 2D NMR data of PhenDC3-yne. 18
Supplementary Figure 7. HRMS data of PhenDC3-btn. 19
Supplementary Figure 8. NMR data of Dox-btn1. 20
Supplementary Figure 9. NMR and HRMS data of Dox-btn1. 21
Supplementary Figure 10. NMR data of Dox-btn2. 22
Supplementary Figure 11. NMR and HRMS data of Dox-btn2. 23
Supplementary Table 1: Sequencing reads information of JQ1-btn Chem-map .24
Supplementary Table 2: DNA oligomers used in the FRET melting assays..........25
Supplementary Table 3: ΔT_m caused by small molecule ligands at 1 μM in FRET melting assays 26
Reference 27

Chemical synthesis

Chemicals and reagents were purchased from Sigma-Aldrich, MedChemExpress, and Fluorochem. All organic solvents were distilled by standard purification methods before use or purchased as anhydrous from Sigma-Aldrich. All reactions were performed in oven-dried glassware under argon unless otherwise stated. NMR spectra were recorded on a Bruker 400 MHz Advance III HD Spectrometer or a 500 MHz DCH Cryoprobe Spectrometer operating at 400 and 500 MHz for ^{1}H NMR and 126 MHz for ^{13}C NMR respectively in DMSO-d_6, and analysed in software MestReNova 14.2.3. NMR data are reported as follows: chemical shifts in parts per million (ppm) referring to the solvent residual peak, multiplicities (s = singlet, d = doublet, t = triplet, q = quartet; m = multiplet, br = broad) and coupling constant (*J*) values in Hz. LC-MS was performed on an Amazon ESI-MS (Bruker) connected to a Dionex UltiMate 3000 UHPLC system (Thermo Fisher Scientific). High-resolution mass spectra (HRMS) were obtained from a Waters Vion IMS QTof spectrometer. Flash column chromatography was performed using CombiFlash Rf (Teledyne ISCO) with C18 puriFlash columns (Interchim). Di-boc protected Pyridostatin (PDS) and PhenDC3-alkyne were prepared according to the previously reported procedures [1, 2]. Commercially available small molecule probes and linkers include (+) JQ-1 carboxylic acid (MedChemExpress, HY-78695) [3], doxorubicin•HCl (ApexBio, A1832), biotin-PEG2-amine (Bachem AG, 4065553.0050), biotin-PEG4-OSu (Sigma-Aldrich, QBD10200), biotin-PEG3-azide (Sigma-Aldrich), and biotin-PEG4-amine (MedChemExpress, HY-140895).

Synthesis of JQ1-btn

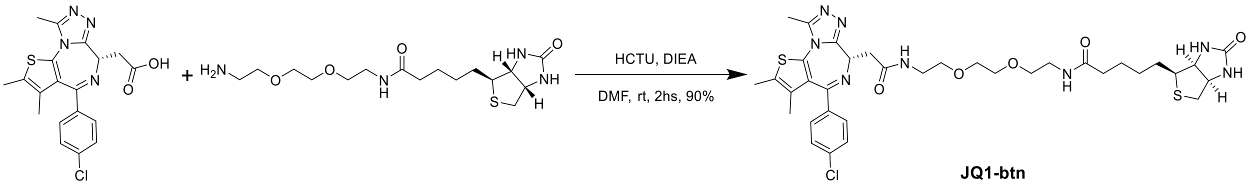

JQ1 (**1**, 10 mg, 25 µmol) and HCTU (12.4 mg, 30 µmol) were dissolved in 0.5 mL anhydrous DMF. Biotin-PEG2-NH2 (11.2 mg, 30 µmol) was added to the mixture followed by the addition of DIEA (23 µL, 125 µmol), and the reaction was stirred for 2 h in room temperature under argon. The solvent was evaporated to dryness *in vacuo* and the product was purified using flash column chromatography (C18 column, gradient elution: water (0.1% (v/v) TFA) to MeCN (0.1% (v/v) TFA) over 30 min at a flow rate of 18 mL/min). Solvents were removed through freeze-drying to obtain the product as a white powder (**JQ1-btn**, 17 mg, yield 90%, Supplementary Fig. 1 and 2).

^{1}H NMR (500 MHz, DMSO) δ 8.27 (t, *J* = 5.7 Hz, 1H), 7.82 (t, *J* = 5.7 Hz, 1H), 7.48 (d, *J* = 8.8 Hz, 2H), 7.44 – 7.39 (m, 2H), 6.40 (s, 1H), 4.51 (dd, *J* = 8.0, 6.3 Hz, 1H), 4.31 – 4.25 (m, 1H), 4.10 (dd, *J* = 7.7, 4.4 Hz, 1H), 3.51 (tt, *J* = 5.3, 2.8 Hz, 4H), 3.44 (t, *J* = 5.9 Hz, 2H), 3.39 (t, *J* = 6.0 Hz, 2H), 3.33 – 3.12 (m, 7H), 3.07 (ddd, *J* = 8.6, 6.2, 4.4 Hz, 1H), 2.79 (dd, *J* = 12.4, 5.1 Hz, 1H), 2.59 (s, 3H), 2.55 (d, *J* = 12.4 Hz, 1H), 2.42 – 2.38 (m, 3H), 2.04 (t, *J* = 7.4 Hz, 2H), 1.61 (s, 3H), 1.59 – 1.53 (m, 1H), 1.51 – 1.40 (m, 3H), 1.27 (dtd, *J* = 15.6, 8.8, 5.9 Hz, 2H). ^{13}C NMR (126 MHz, DMSO) δ 172.59, 170.07, 163.61, 163.15, 155.50, 150.42, 137.08, 135.76, 132.65, 131.34, 130.66, 130.33, 130.06, 128.92, 70.03, 70.00, 69.65, 69.63, 61.48, 59.64, 55.86, 54.20, 40.13, 40.05, 39.96, 39.88, 39.79, 39.08, 38.89, 37.85, 35.54, 28.64, 28.48, 25.70, 14.51, 13.14, 11.74. HRMS (ESI-QTof) m/z: $[M + H]^+$ calculated for $C_{35}H_{46}ClN_8O_5S_2^+$: 757.3660; found, 757.2702.

Synthesis of PDS-btn

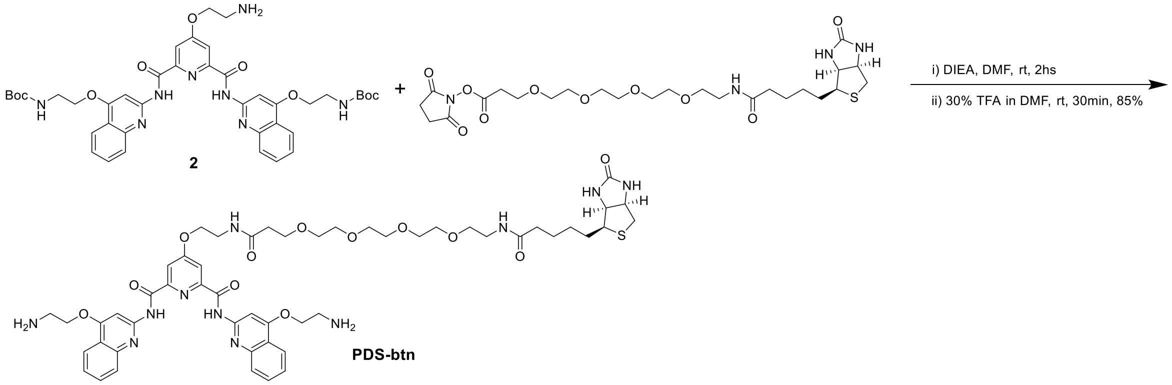

The synthesis of di-boc protected PDS (**2**) has been reported previously[4]. Di-boc protected PDS (**2**, 20 mg, 25 µmol) and biotin-PEG4-OSu (17.6mg, 30µmol) were dissolved in 0.5 mL anhydrous DMF. DIEA (23 µL, 125 µmol) was added, and the reaction was stirred for 2 h in room temperature under argon. The solvent was evaporated to dryness *in vacuo*. The crude was dissolved in 1 mL 30% TFA in DMF (v/v) and stirred for 30 min in room temperature and the solvent was evaporated to dryness *in vacuo*. The product was purified using flash column chromatography (C18 column, gradient elution: water (0.1% (v/v) TFA) to MeCN (0.1% (v/v) TFA) over 30min at a flow rate of 18 mL/min)). Solvents were removed through freeze-drying to obtain the product as a white powder (**PDS-btn**, 22.7 mg, yield 85%, Supplementary Fig. 3 and 4).

^{1}H NMR (500 MHz, DMSO) δ 12.08 (s, 2H), 8.42 (dd, *J* = 8.3, 1.5 Hz, 2H), 8.18 (t, *J* = 5.6 Hz, 1H), 8.16 – 8.07 (m, 8H), 7.96 – 7.92 (m, 2H), 7.91 (s, 2H), 7.79 (ddd, *J* = 8.4, 5.6, 1.6 Hz, 3H), 7.55 (ddd, *J* = 8.2, 6.8, 1.2 Hz, 2H), 6.39 (s, 1H), 6.34 (s, 1H), 4.49 (t, *J* = 5.0 Hz, 4H), 4.32 (t, *J* = 5.5 Hz, 2H), 4.27 (dd, *J* = 7.7, 4.8 Hz, 1H), 4.09 (dd, *J* = 7.8, 4.5 Hz, 1H), 3.60 (t, *J* = 6.4 Hz, 3H), 3.56 – 3.47 (m, 13H), 3.34 (t, *J* = 5.9 Hz, 4H), 3.14 (q, *J* = 5.9 Hz, 2H), 3.05 (ddd, *J* = 8.6, 6.2, 4.4 Hz, 1H), 2.78 (dd, *J* = 12.5, 5.1 Hz, 1H), 2.58 – 2.51 (m, 1H), 2.35 (t, *J* = 6.4 Hz, 2H), 2.03 (t, *J* = 7.5 Hz, 2H), 1.57

(ddt, J = 12.3, 9.6, 6.0 Hz, 1H), 1.44 (ddt, J = 13.9, 10.0, 6.2 Hz, 3H), 1.26 (h, J = 8.1 Hz, 2H). ^{13}C NMR (126 MHz, DMSO) δ 172.60, 171.06, 167.59, 163.74, 163.16, 162.14, 152.76, 151.49, 147.46, 131.29, 127.19, 124.99, 123.15, 119.47, 115.65, 112.57, 95.47, 70.20, 70.14, 70.11, 69.98, 69.58, 67.95, 67.17, 65.61, 61.48, 59.64, 55.86, 40.22, 40.13, 40.05, 39.96, 39.88, 39.79, 38.87, 38.69, 36.49, 35.52, 28.63, 28.48, 25.70. HRMS (ESI-QTof) m/z: $[M + H]^+$ calculated for $C_{52}H_{68}N_{11}O_{12}S_2^+$: 1070.2330; found, 1070.4765, 535.7427.

Synthesis of PhenDC3-btn

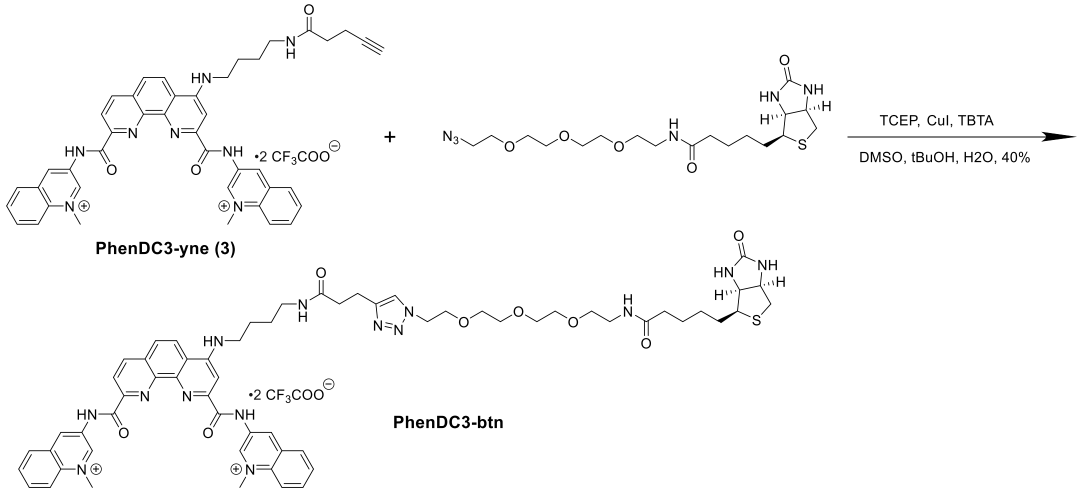

PhenDC3-yne (**3**) was prepared by following reports by Lefebvre *et al.* [2] and characterized it using NMR and HRMS (Supplementary Fig. 5 and 6). ^{1}H NMR (400 MHz, DMSO) δ 12.15 (s, 2H), 10.30 (d, J = 2.8 Hz, 2H), 9.88 (s, 2H), 8.87 (d, J = 8.4 Hz, 1H), 8.68 (d, J = 8.3 Hz, 1H), 8.62 (d, J = 9.4 Hz, 1H), 8.56 – 8.47 (m, 4H), 8.25 (s, 2H), 8.21 (d, J = 9.5 Hz, 1H), 8.07 (t, J = 6.8 Hz, 3H), 7.98 (s, 1H), 7.70 (s, 1H), 4.73 (s, 3H), 4.72 (s, 3H), 3.51 (s, 2H), 3.18 (d, J = 6.0 Hz, 2H), 2.75 (t, J = 2.6 Hz, 1H), 2.39 – 2.34 (m, 2H), 2.28 (t, J = 7.0 Hz, 2H), 1.80 (s, 2H), 1.62 (s, 2H). ^{13}C NMR (101 MHz, DMSO) δ 170.2, 164.2, 163.7, 152.5, 148.0, 145.8, 145.7, 145.8, 145.7, 138.8, 135.5, 135.4, 134.7, 134.5, 133.9, 133.8, 132.8, 131.0, 130.3, 130.0, 129.9, 129.2, 125.3,

122.9, 121.6, 119.4, 119.2, 99.7, 83.8, 71.3, 46.0, 42.6, 38.2, 34.3, 27.0, 25.1, 14.3; HRMS (ESI$^+$) calculated for $[C_{45}H_{40}N_8O_5F_3]^+$: 829.3068, m/z found: 829.3057.

PhenDC3-yne (**3**, 5.4 mg, 6.5 µmol) and Biotin-PEG3-azide (6 mg, 13 µmol) were suspended in 75 µL DMSO. Tris(2-carboxyethyl)phosphine (TCEP, 0.33 mmol, freshly prepared 100 mM solution in water) was added, followed by copper(II) sulphate pentahydrate (0.33 mmol, freshly prepared 10 mM in water). Tris(benzyltriazolylmethyl)amine (TBTA, 0.33 mmol, stock solution 40 mM in 1:1 water/*tert*-butanol mixture) was added to the solution and stirred for 3 h at room temperature under argon. The mixture was purified using flash column chromatography (C18 column, gradient elution: water (0.1% (v/v) TFA) to MeCN (0.1% (v/v) TFA) over 30 min at a flow rate of 18 mL/min. Solvents were removed through freeze-drying to obtain the product as a yellow powder (**PhenDC3-btn**, 2.9 mg, yield 41%, Supplementary Fig. 7). HRMS (ESI-QTof) m/*z*: $[M]^{2+}$ calculated for $C_{61}H_{72}N_{14}O_8S^{2+}$: 580.2683; found, 580.2676, 387.1818.

Synthesis of Dox-btn1

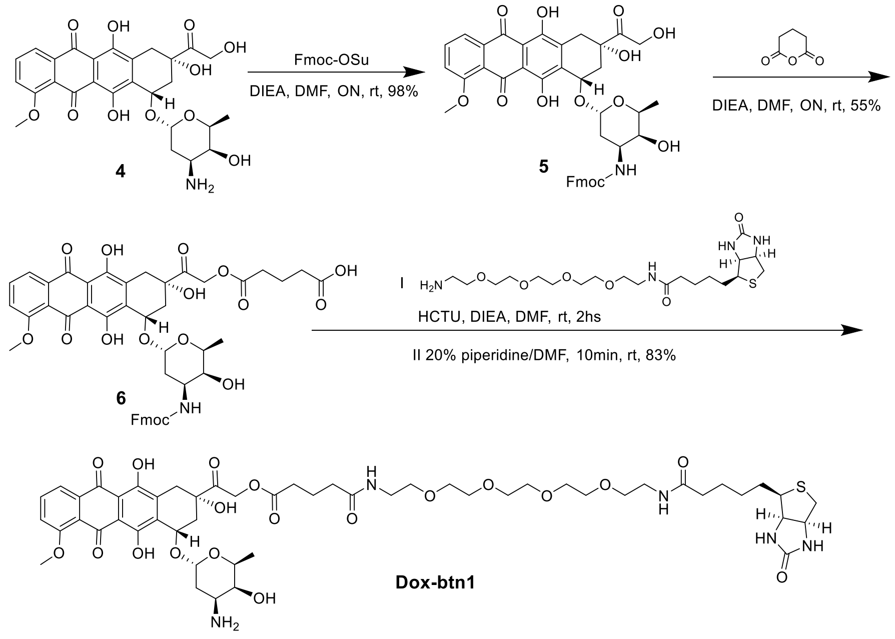

Following reports by Tjandra *et al.* [5], doxorubicin·HCl (**4**, 29 mg, 50 µmol) was dissolved in 1 mL anhydrous DMF. Fmoc *N*-hydroxysuccinimide ester (Fmoc-OSu, 26 mg, 75 µmol) was dissolved in 1 mL DMF and then added to the solution with constant stirring. DIEA (60 µL, 340 µmol) was added dropwise to the mixture upon which a dark red solution formed. The mixture was stirred at room temperature under argon and protected from light. The reaction stopped after 4 h. DMF was removed *in vacuo*, and the remaining red oil was added with 0.1% (v/v) aqueous TFA to give red crystals and washed with cold Et_2O. The product doxorubicin-Fmoc (**5**) was then dried *in vacuo* and collected as red crystals (38 mg, 98%). This intermediate (**5**, 38 mg, 0.05 mmol) was redissolved in 5 mL anhydrous DMF and reacted with glutaric anhydride (53 mg, 0.25 mmol). DIEA (18 µL, 0.1 mmol) was added dropwise to the reaction mixture to form a dark red solution. The mixture was protected from light and stirred overnight at room temperature under nitrogen atmosphere. After 24 hr, the reaction mixture was concentrated, and the residual red oil was triturated with 0.1% (v/v) aqueous TFA to produce a red solid powder. This crude product was purified using C18 flash column with MeCN (B) and 0.1% formic acid in water (A) as eluent and obtained red powder (**6**, 24 mg, yield 55%). *N*-Fmoc-doxorubicin-*O*-hemiglutarate (**6**) (10 mg, 11 µmol) was dissolved in 0.5 ml anhydrous DMF. HCTU (5.6 mg, 13 µmol) and DIEA (3.8 µL, 22 µmol) were added to the solution. The mixture was stirred for 2 h under argon atmosphere. The solvent was evaporated to dryness *in vacuo*. The intermediate proceeded to Fmoc deprotection and was resuspended in 1 mL 20% piperidine/DMF (v/v) solution and stirred for 10 min at room temperature. The solvent was evaporated to dryness *in vacuo*. The product was purified using flash column chromatography (C18 column, gradient elution: water (0.1% (v/v) TFA) to MeCN (0.1% (v/v) TFA)

over 30 min at a flow rate of 18 mL/min). Solvents were removed through freeze-drying to obtain the product as a red powder (**Dox-btn1**, 10.1 mg, yield 83%, Supplementary Fig. 8 and 9).

^{1}H NMR (500 MHz, DMSO) δ 14.05 (s, 1H), 13.27 (s, 1H), 7.96 – 7.93 (m, 2H), 7.86 (t, *J* = 5.6 Hz, 1H), 7.82 (t, *J* = 5.7 Hz, 1H), 7.74 (d, *J* = 5.3 Hz, 3H), 7.71 – 7.66 (m, 1H), 6.40 (s, 1H), 6.35 (s, 1H), 5.59 (s, 1H), 5.46 (d, *J* = 6.3 Hz, 1H), 5.32 – 5.28 (m, 1H), 5.24 (d, *J* = 17.8 Hz, 1H), 5.15 (d, *J* = 17.8 Hz, 1H), 4.98 (dd, *J* = 5.5, 2.9 Hz, 1H), 4.30 (dd, *J* = 7.8, 5.0 Hz, 1H), 4.21 (q, *J* = 6.3 Hz, 1H), 4.12 (ddd, *J* = 7.8, 4.4, 1.9 Hz, 1H), 4.00 (s, 4H), 3.55 (d, *J* = 5.6 Hz, 1H), 3.39 (q, *J* = 5.9 Hz, 6H), 3.18 (p, *J* = 6.0 Hz, 5H), 3.13 – 3.07 (m, 3H), 3.05 (s, 1H), 2.93 (d, *J* = 18.2 Hz, 1H), 2.81 (dd, *J* = 12.4, 5.1 Hz, 1H), 2.57 (d, *J* = 12.4 Hz, 1H), 2.40 (t, *J* = 7.5 Hz, 2H), 2.28 (d, *J* = 14.4 Hz, 1H), 2.14 (t, *J* = 7.4 Hz, 2H), 2.10 (d, *J* = 5.6 Hz, 1H), 2.05 (t, *J* = 7.4 Hz, 3H), 1.90 (td, *J* = 12.7, 3.7 Hz, 1H), 1.77 (p, *J* = 7.6 Hz, 3H), 1.69 (dd, *J* = 12.2, 4.4 Hz, 1H), 1.60 (ddd, *J* = 15.9, 10.1, 4.7 Hz, 1H), 1.54 – 1.39 (m, 4H), 1.29 (dt, *J* = 15.7, 7.7 Hz, 3H), 1.19 – 1.14 (m, 5H). ^{13}C NMR (126 MHz, DMSO) δ 207.82, 186.71, 186.61, 172.13, 172.10, 171.54, 162.69, 160.86, 155.94, 154.44, 136.38, 135.10, 134.83, 133.85, 120.05, 119.85, 119.09, 110.86, 110.79, 99.27, 75.10, 69.78, 69.72, 69.55, 69.16, 69.10, 66.20, 66.14, 65.41, 61.02, 59.18, 56.66, 55.41, 46.66, 45.75, 39.69, 39.61, 39.52, 39.44, 38.45, 38.43, 36.11, 35.08, 34.13, 32.58, 31.87, 28.19, 28.03, 25.25, 20.65, 16.62, 8.64. HRMS (ESI-QTof) *m/z*: $[M + H]^+$ calculated for $C_{52}H_{73}N_5O_{19}S^+$: 1102.2160; found, 1102.4580.

Synthesis of Dox-btn2

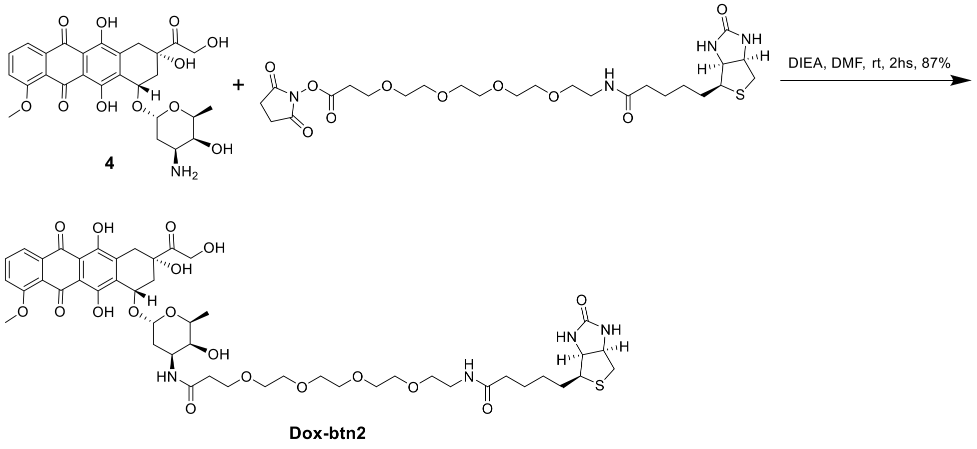

To a 5 mL flask, doxorubicin·HCl (**4**, 20 mg, 36 μmol) and biotin-PEG4-OSu (22 mg, 36 μmol) were dissolved in 1 mL anhydrous DMF. DIEA (7.1 μL, 55 μmol) was added, and the reaction was stirred for 2 h in room temperature under argon. The solvent was evaporated to dryness *in vacuo*. The product was purified using flash column chromatography (C18 column, gradient elution: water (0.1% (v/v) TFA) to MeCN (0.1% (v/v) TFA) over 30 min at a flow rate of 18 mL/min). Solvents were removed through freeze-drying to obtain the product as a red powder (**Dox-btn2**, 32 mg, yield 87%, Supplementary Fig. 10 and 11).

^{1}H NMR (500 MHz, DMSO) δ 14.04 (s, 1H), 13.28 (s, 1H), 7.94 – 7.87 (m, 2H), 7.79 (t, *J* = 5.7 Hz, 1H), 7.69 – 7.61 (m, 1H), 7.55 (d, *J* = 8.1 Hz, 1H), 6.39 (s, 1H), 6.33 (s, 1H), 5.45 (s, 1H), 5.21 (d, *J* = 3.7 Hz, 1H), 4.94 (dd, *J* = 5.6, 3.5 Hz, 1H), 4.55 (s, 2H), 4.31 – 4.25 (m, 1H), 4.14 (q, *J* = 6.7 Hz, 1H), 4.10 (dd, *J* = 7.8, 4.4 Hz, 1H), 3.98 (s, 3H), 3.95 (s, 1H), 3.57 (s, 4H), 3.51 (td, *J* = 6.6, 1.7 Hz, 4H), 3.42 (ddt, *J* = 8.5, 5.5, 3.6 Hz, 6H), 3.38 – 3.30 (m, 4H), 3.14 (q, *J* = 5.9 Hz, 2H), 3.10 – 2.99 (m, 1H), 2.97 (d, *J* = 5.0 Hz, 2H), 2.79 (dd, *J* = 12.4, 5.1 Hz, 1H), 2.61 – 2.52 (m, 1H), 2.41 – 2.08 (m, 5H), 2.03 (t, *J* = 7.4 Hz, 2H), 1.81 (td, *J* = 12.9, 4.0 Hz, 1H), 1.58 (ddd, *J* = 20.6, 10.7, 6.1 Hz, 1H), 1.52 – 1.37 (m, 4H), 1.27 (dq, *J* = 14.3, 7.6 Hz, 2H), 1.11 (d, *J* = 6.5 Hz,

3H). ^{13}C NMR (126 MHz, DMSO) δ 207.82, 186.71, 186.61, 172.13, 172.10, 171.54, 162.69, 160.86, 157.75, 157.51, 155.94, 154.44, 136.38, 135.10, 134.83, 133.85, 120.05, 119.85, 119.09, 110.86, 110.79, 99.27, 75.10, 69.78, 69.72, 69.55, 69.16, 69.10, 66.20, 66.14, 65.41, 61.02, 59.18, 56.66, 55.41, 46.66, 45.75, 38.45, 38.43, 36.11, 35.08, 34.13, 32.58, 31.87, 28.19, 28.03, 25.25, 20.65, 16.62, 8.64. HRMS (ESI-QTof) m/z: $[M + H]^+$ calculated for $C_{48}H_{65}N_4O_{18}S^+$: 1017.1100; found, 1017.4043.

Supplementary Technical Discussion:

Replication

We employed five technical replicates to assess the experimental reproducibility. In addition, we employed two or three biological replicates depending on the expected reproducibility of biological conditions (higher variability for small molecule perturbation than biological replication of independent cell culture passage numbers) [6]. Given the excellent technical reproducibility observed for all the tested small molecule probes (see Fig. 1, Extended Date Figure S2–S4), we suggest that technical replication can be substantially reduced. The number of biological replicates largely depends on the experimental questions and expected variability across investigated conditions.

Site discovery

Due to the sparseness of background, we employed the Sparse Enrichment Analysis for CUT&RUN (SEACR) package for peak calling, which has been specifically designed to call peaks from chromatin profiling data with very low backgrounds (i.e. regions with no or very low read coverage) [7]. We opted for standard peak calling parameters employed in CUT&Tag and CUT&RUN (see Technical Figure 1) [8]. In addition, high confidence peaks across replicates were called high stringency (present in 3/5 technical replicates within biological replicates followed by intersecting of all biological replicates). It is noteworthy that peak numbers are dependent on the respective peak calling thresholds and sequencing depth (see Table S1), and our stringent approach likely discards a considerable amount of valuable information on lower populated binding sites. Conversely, in the case of low-abundance targets or lower affinity probes that require stronger signal amplification, the enhanced Tn5 background signal at open chromatin regions may require a more stringent peak calling approach. We advise to use our specified parameters as a starting point and then further optimise based on the respective experimental question.

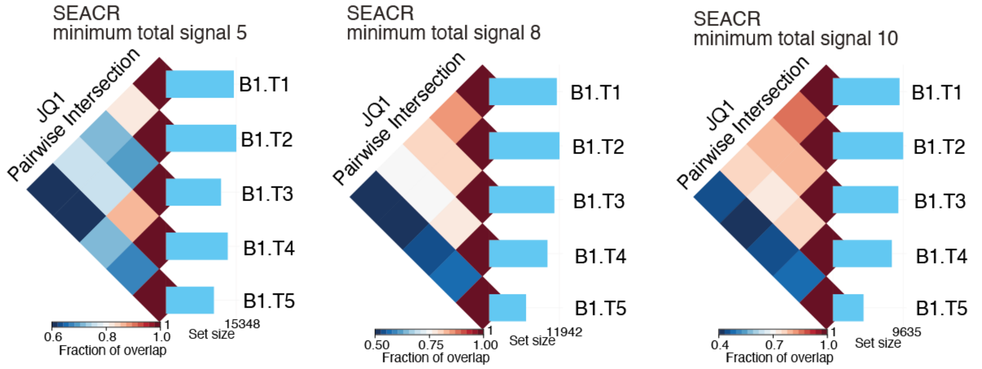

Technical Figure 1. Impact of SEACR peak calling parameters. Pairwise intersection of enriched peaks from JQ1 Chem-map in K562 cells for a 1st biological replicate comprising five technical replicates. Results for different required total signal contained within denoted coordinates (SEACR option "total signal").

a

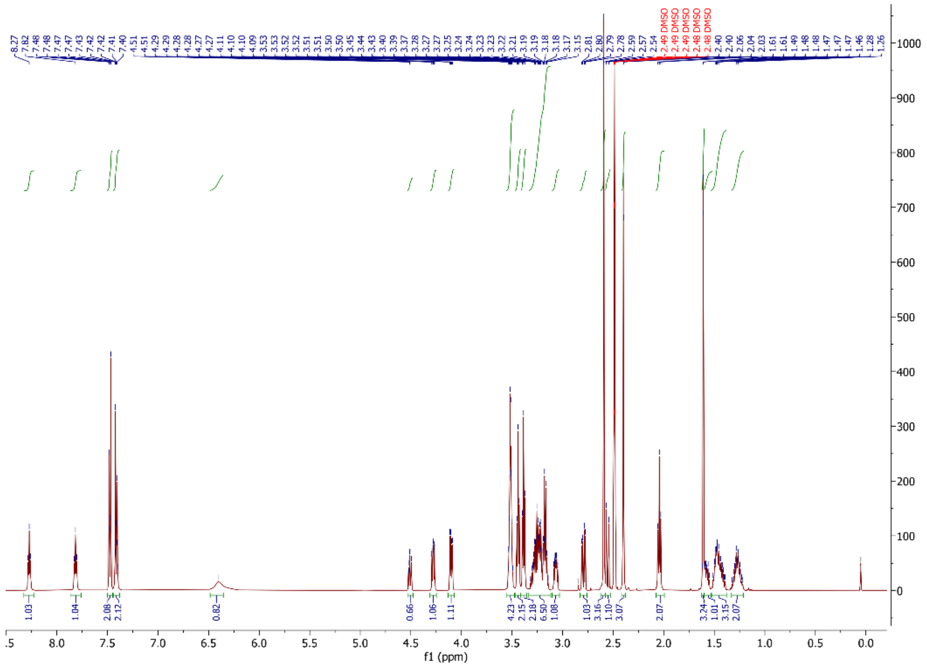

b

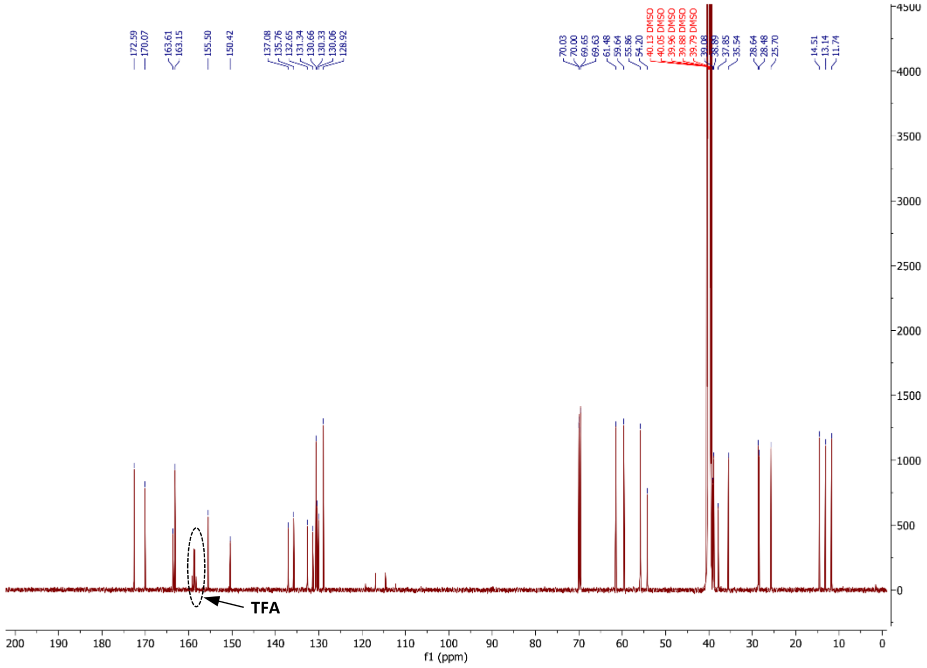

Supplementary Figure 1. NMR data of JQ1-btn.

(**a**) ^{1}H NMR spectrum (400 MHz) in DMSO-d_6 solvent. (**b**) ^{13}C NMR spectrum (126 MHz) in DMSO-d_6 solvent.

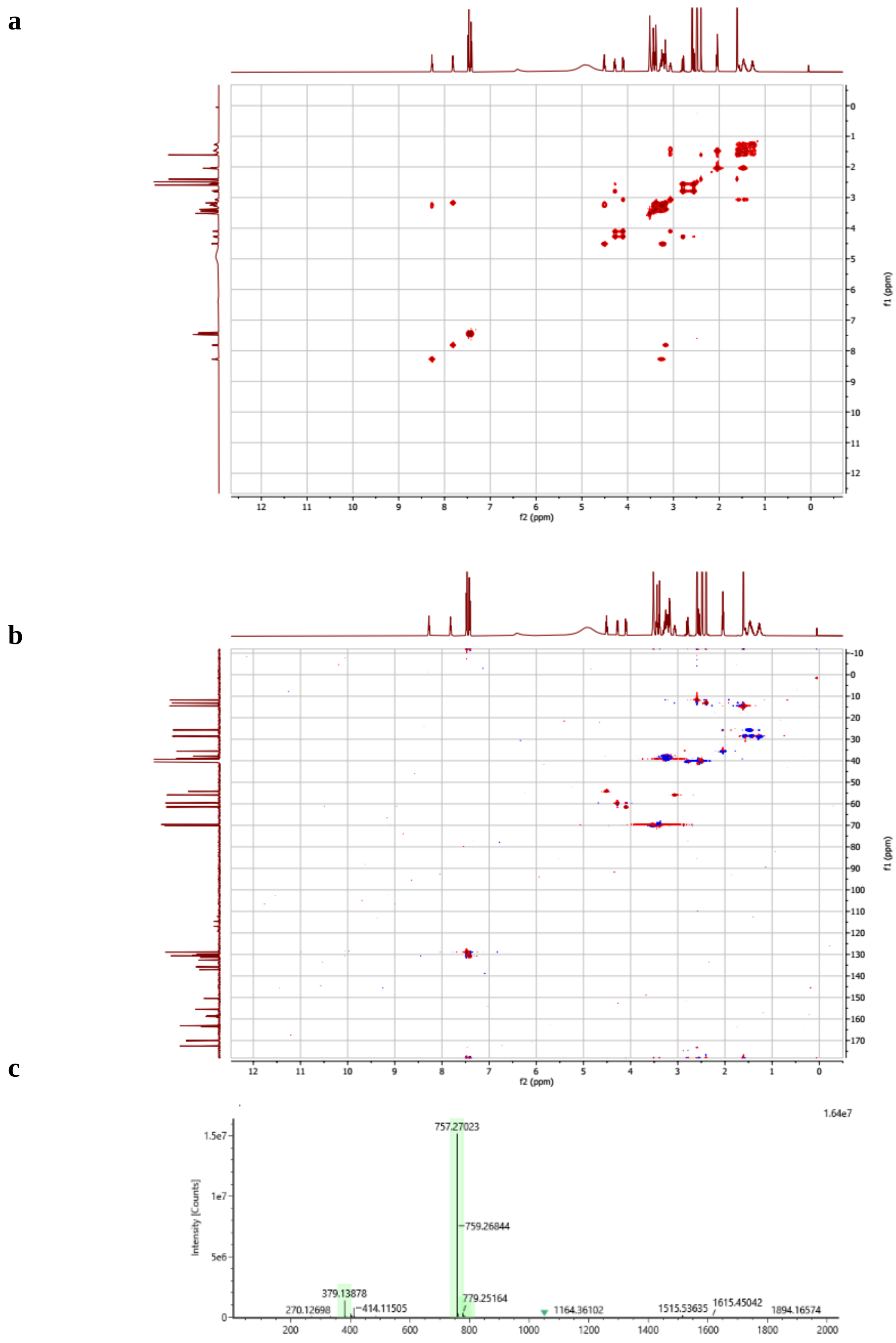

Supplementary Figure 2. NMR and HRMS data of JQ1-btn.

(**a**) COSY NMR spectrum (400 MHz) in DMSO-d_6 solvent. (b) HSQC NMR spectrum (126 MHz) in DMSO-d_6 solvent. (**c**) HRMS spectrum.

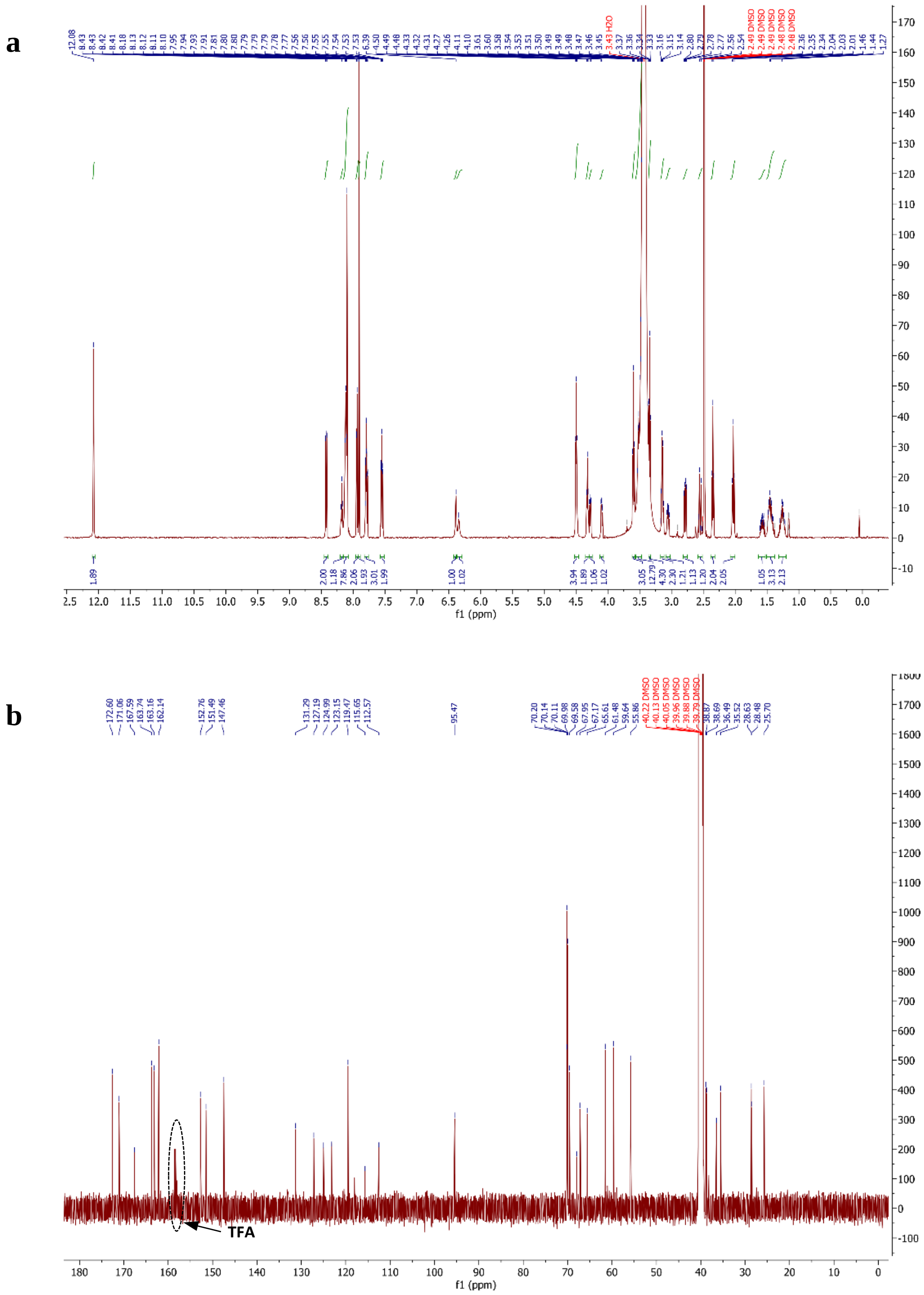

Supplementary Figure 3. NMR data of PDS-btn.

(**a**) ^{1}H NMR spectrum (400 MHz) in DMSO-d_6 solvent. (**b**) ^{13}C NMR spectrum (126 MHz) in DMSO-d_6 solvent.

a

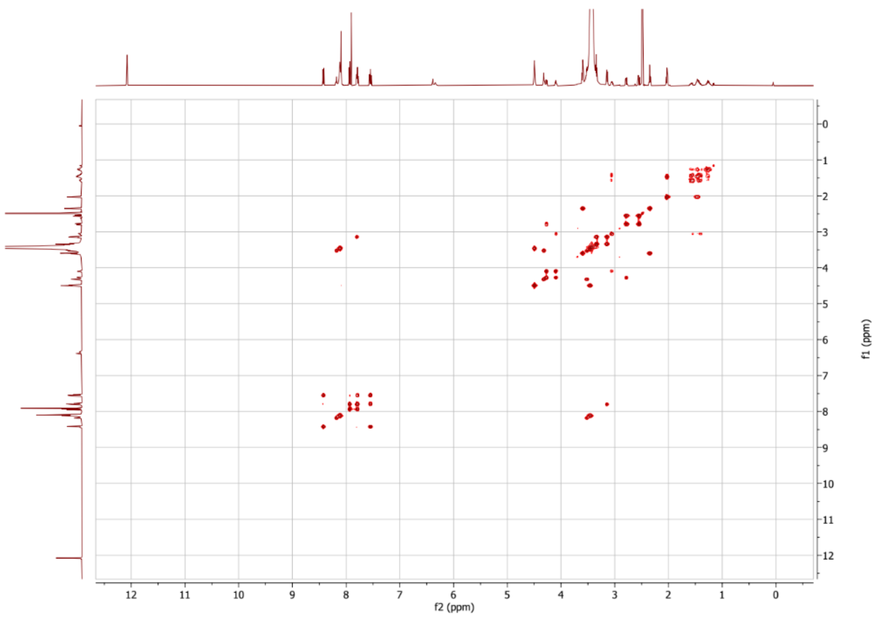

b

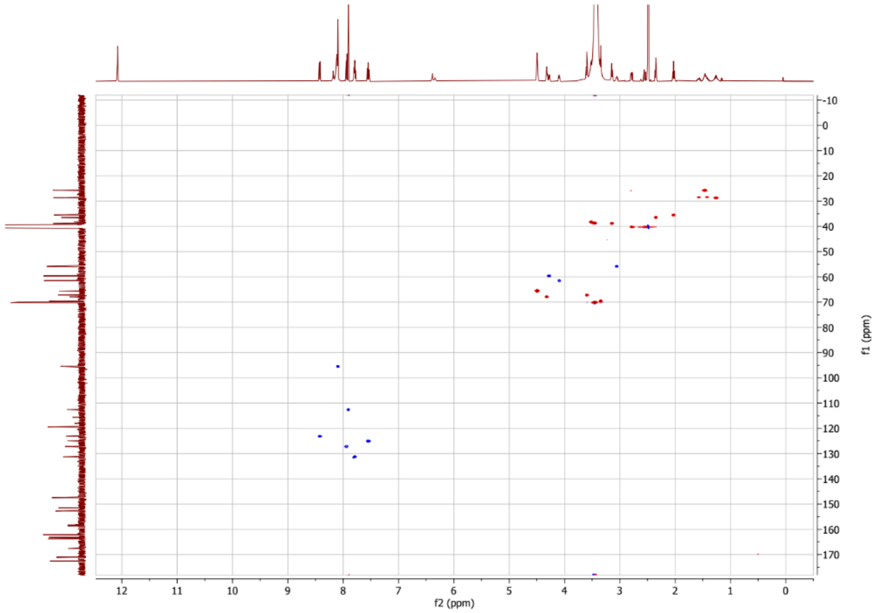

c

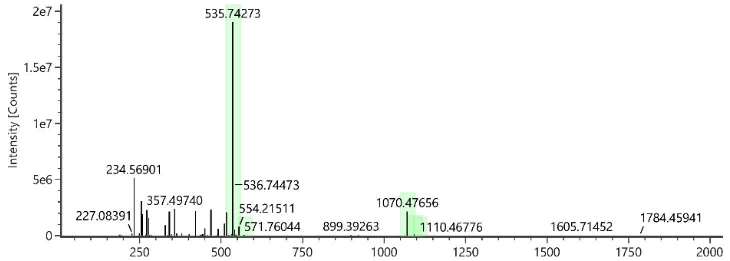

Supplementary Figure 4. NMR and HRMS data of PDS-btn.

(**c**) COSY NMR spectrum (400 MHz) in DMSO-d_6 solvent. (**b**) HSQC NMR spectrum (126 MHz) in DMSO-d_6 solvent. (**c**) HRMS spectrum.

a

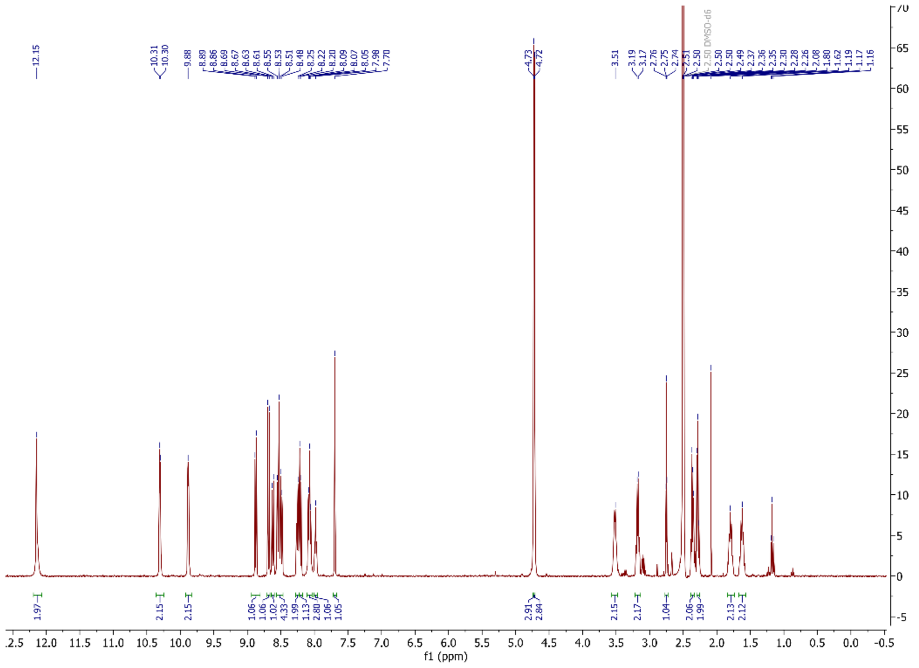

b

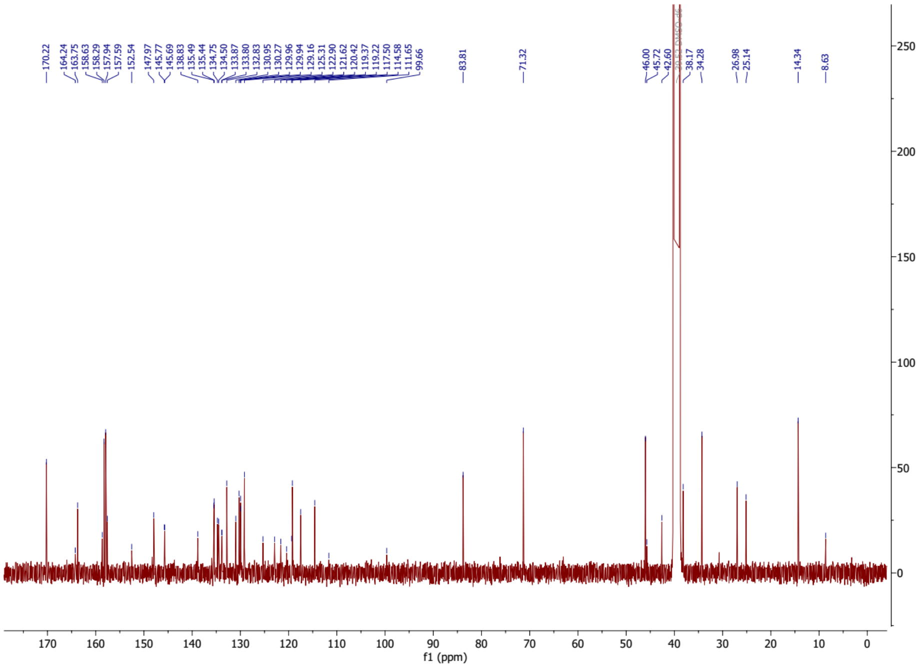

Supplementary Figure 5. NMR data of PhenDC3-yne.
(**a**) ^{1}H NMR spectrum (400 MHz) in DMSO-d_6 solvent. (**b**) ^{13}C NMR spectrum (126 MHz) in DMSO-d_6 solvent.

a

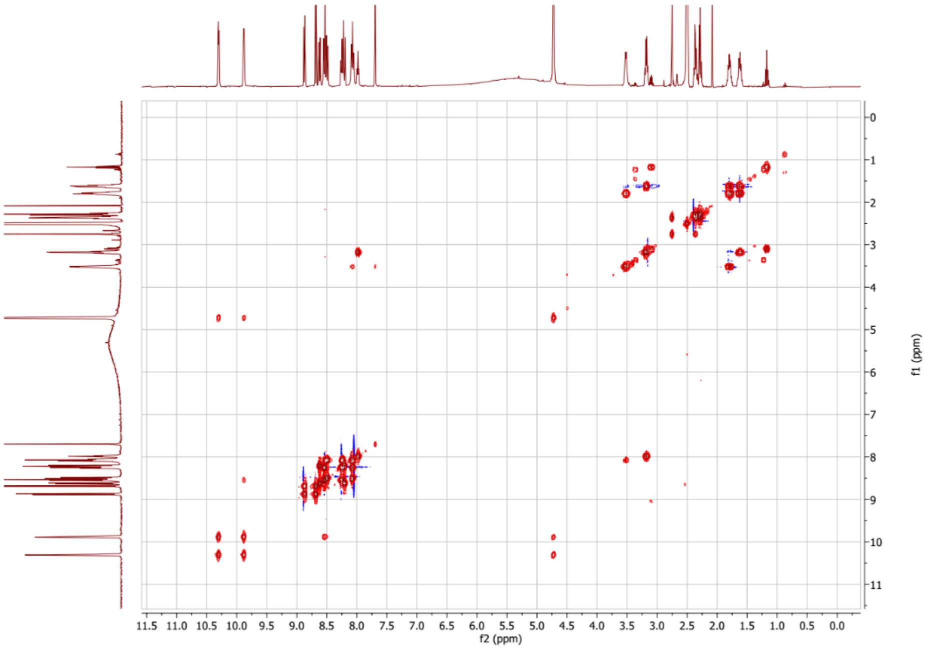

b

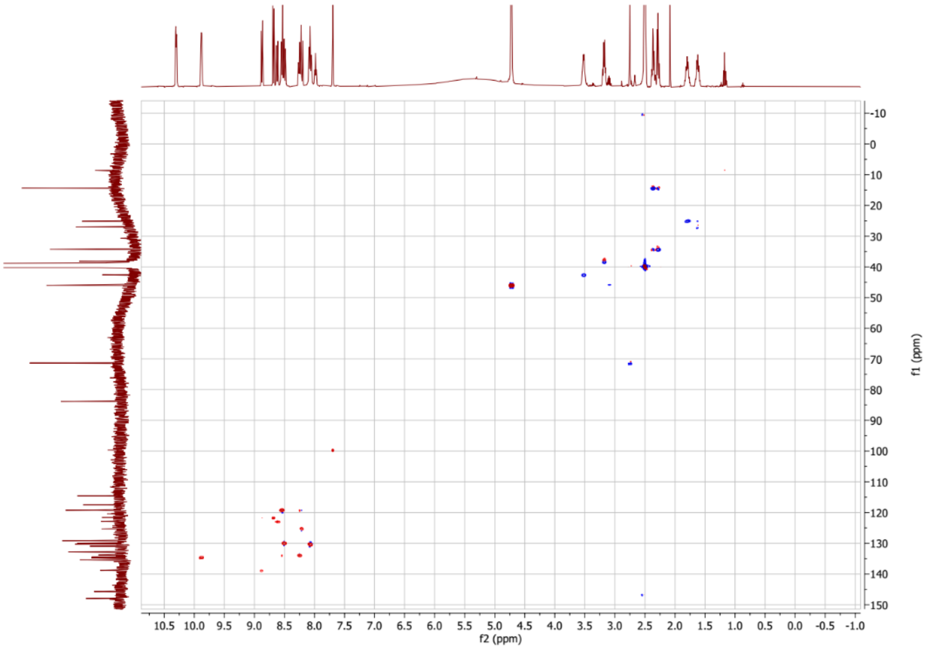

Supplementary Figure 6. 2D NMR data of PhenDC3-yne.

(a) COSY NMR spectrum (400 MHz) in DMSO-d_6 solvent. (**b**) HSQC NMR spectrum (126 MHz) in DMSO-d_6 solvent.

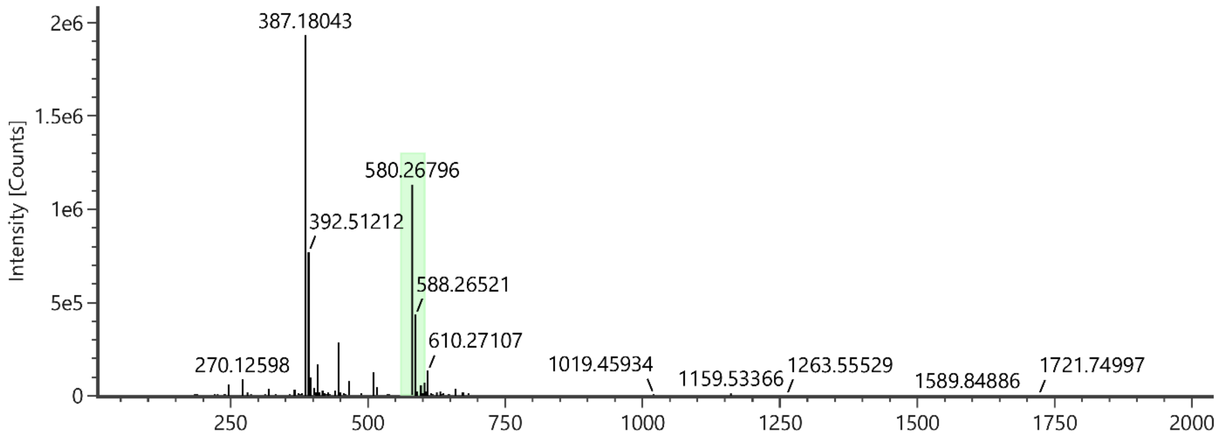

Supplementary Figure 7. HRMS data of PhenDC3-btn.

a

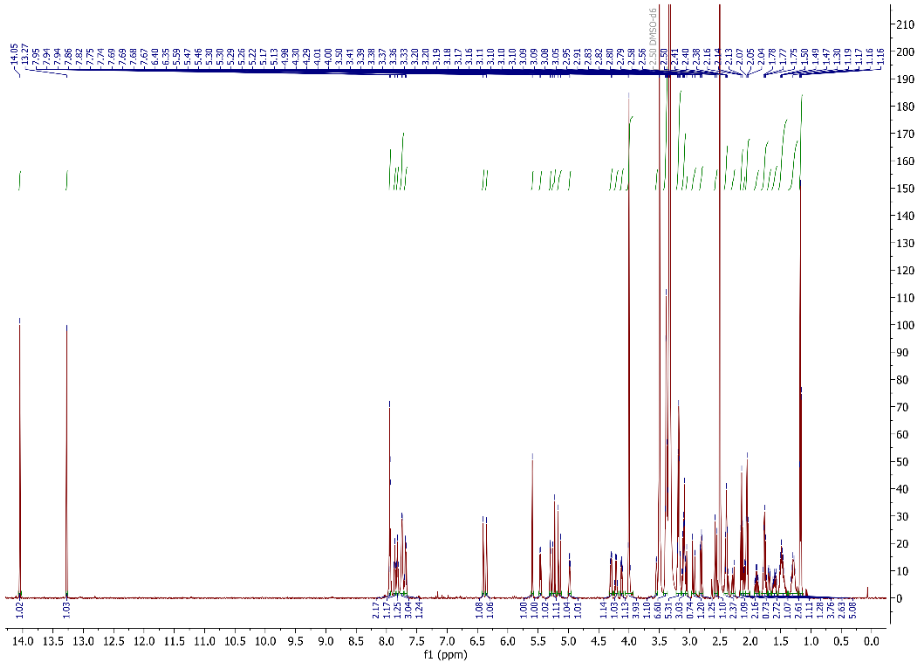

b

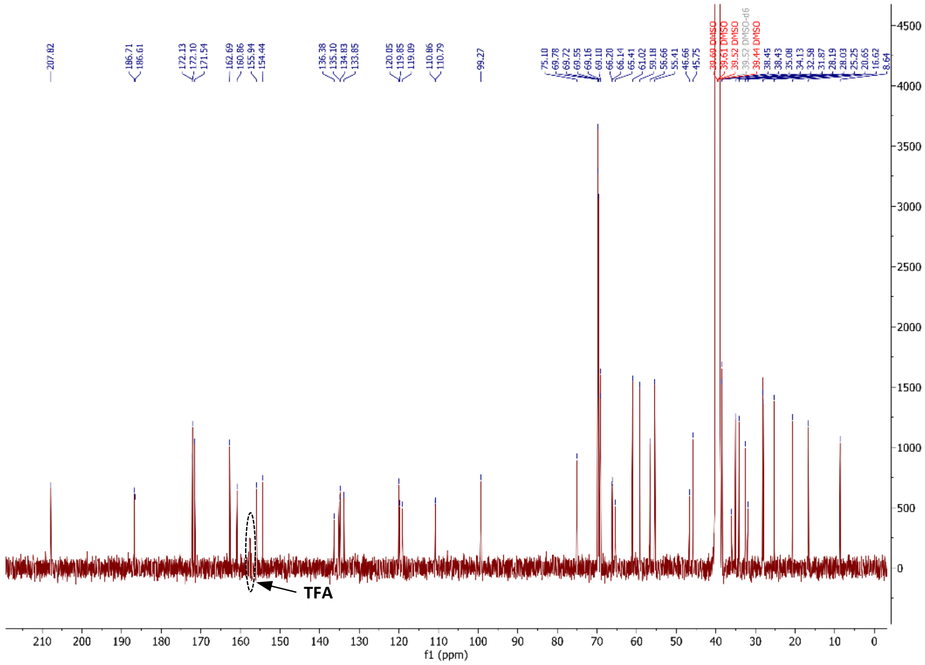

Supplementary Figure 8. NMR data of Dox-btn1.

(**a**) ^{1}H NMR spectrum (400 MHz) in DMSO-d_6 solvent. (**b**) ^{13}C NMR spectrum (126 MHz) in DMSO-d_6 solvent.

a

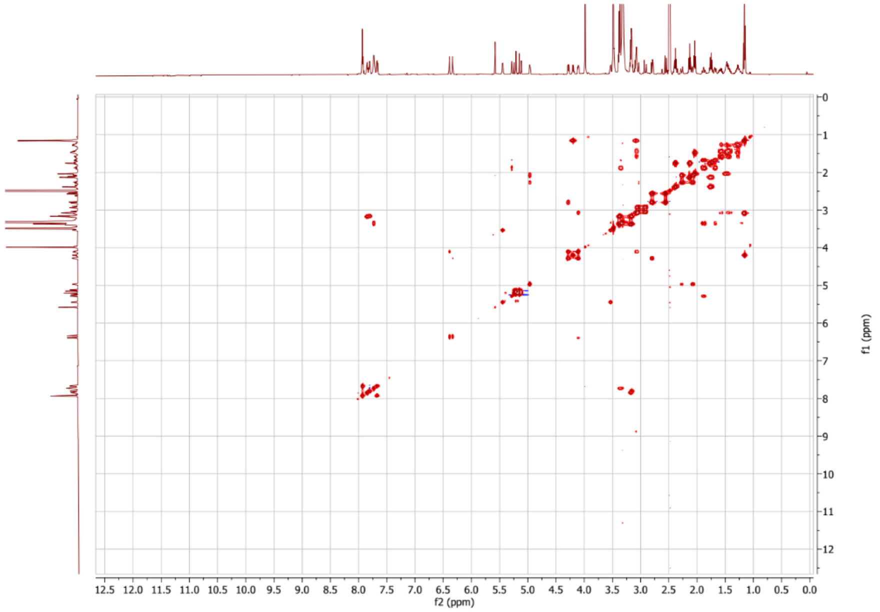

b

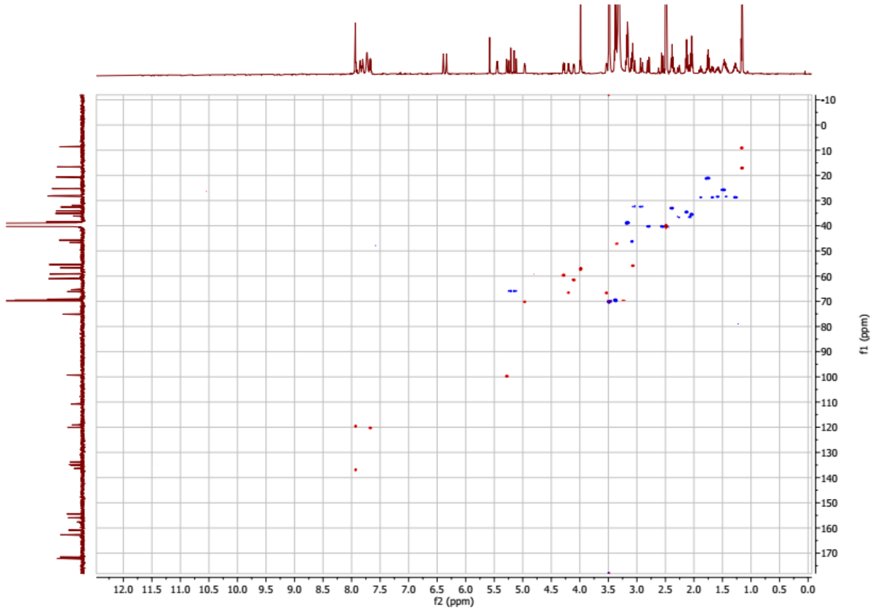

c

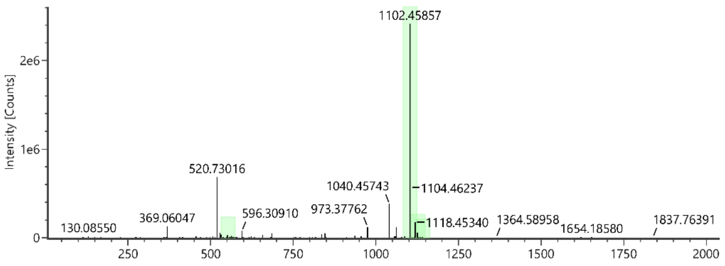

Supplementary Figure 9. NMR and HRMS data of Dox-btn1.

(**a**) COSY NMR spectrum (400 MHz) in DMSO-d_6 solvent. (**b**) HSQC NMR spectrum (126 MHz) in DMSO-d_6 solvent. (**c**) HRMS spectrum.

a

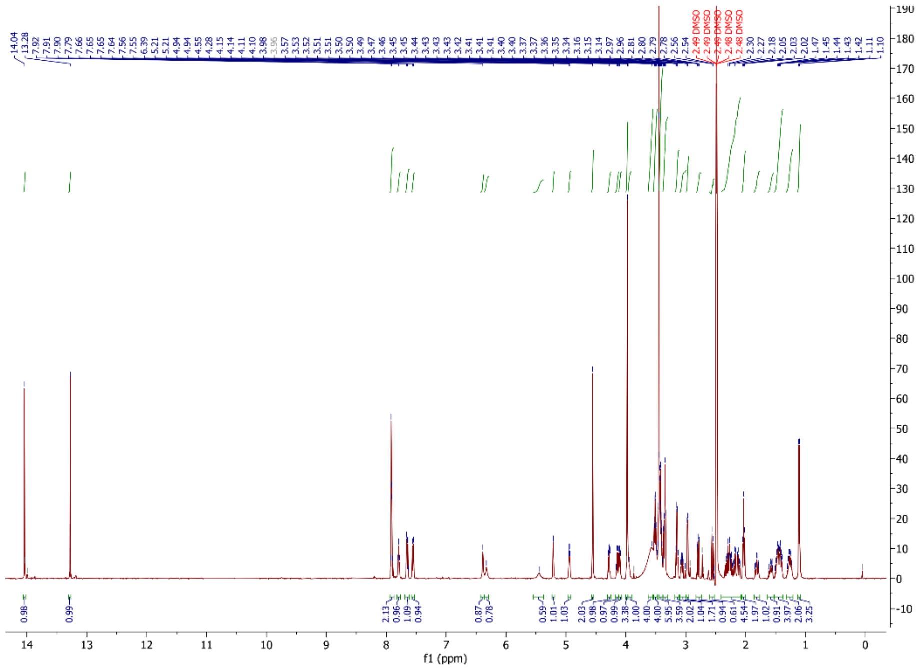

b

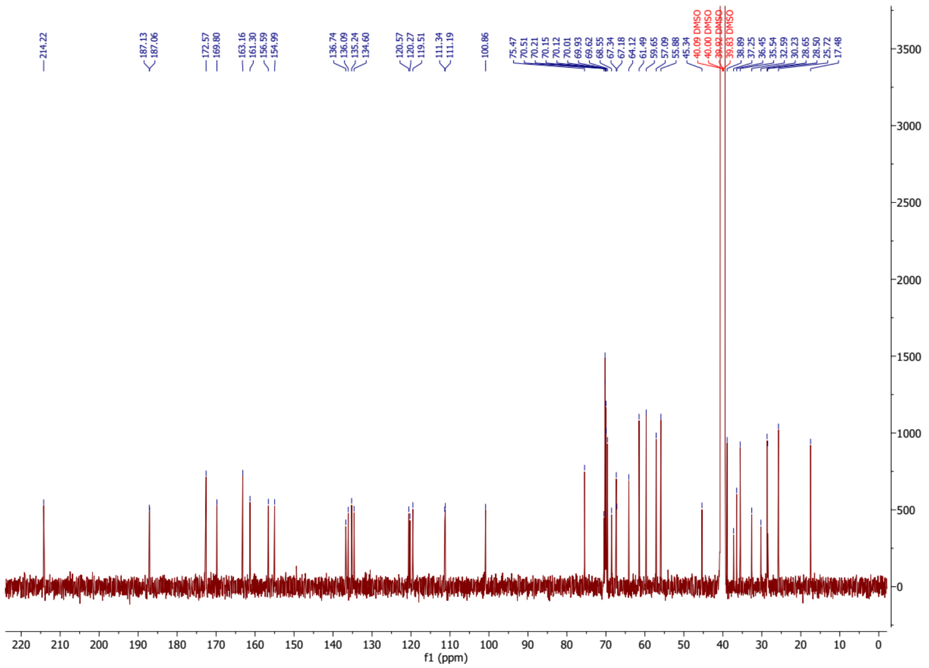

Supplementary Figure 10. NMR data of Dox-btn2.

(**a**) ^{1}H NMR spectrum (400 MHz) in DMSO-d_6 solvent. (**b**) ^{13}C NMR spectrum (126 MHz) in DMSO-d_6 solvent.

a

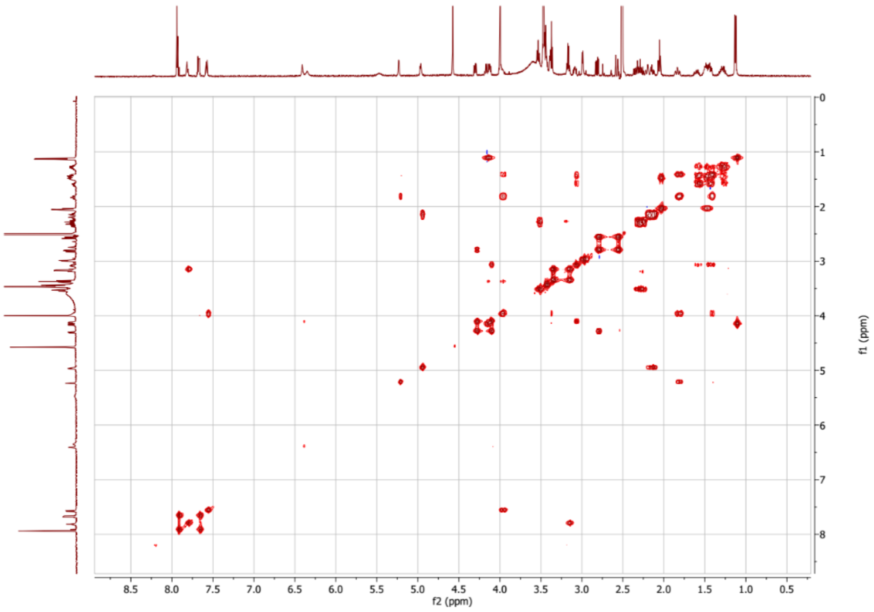

b

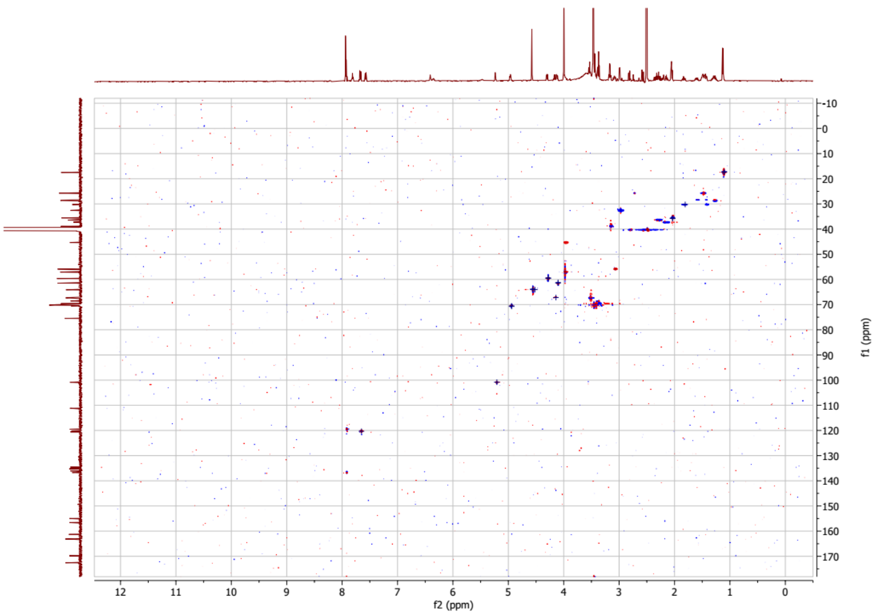

c

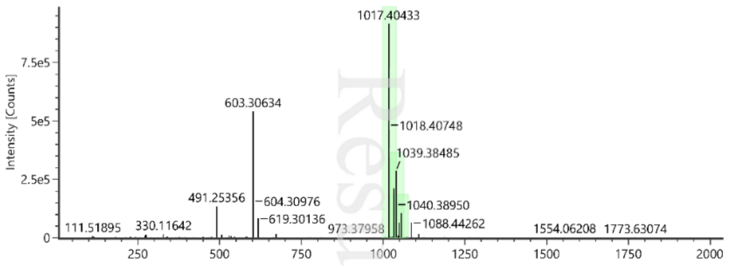

Supplementary Figure 11. NMR and HRMS data of Dox-btn2.

(**a**) COSY NMR spectrum (400 MHz) in DMSO-d_6 solvent. (**b**) HSQC NMR spectrum (126 MHz) in DMSO-d_6 solvent. (**c**) HRMS spectrum.

Supplementary Table 1: Sequencing reads information of JQ1-btn Chem-map

Sample name	Total reads	Mapped reads to hg38	Duplication ratio	Unique reads
SLX-21036_JQ1.pc10.B1.t1	25434498	6389158	0.581472	3049509
SLX-21036_JQ1.pc10.B1.t2	40418390	10445291	0.690318	3390609
SLX-21036_JQ1.pc10.B1.t3	28859174	5566908	0.646032	2125493
SLX-21036_JQ1.pc10.B1.t4	31942803	7494004	0.587691	3502232
SLX-21036_JQ1.pc10.B1.t5	30213398	6962171	0.646498	2653864
SLX-21036_JQ1.pc10.B2.t1	18763127	7780342	0.533406	4364716
SLX-21036_JQ1.pc10.B2.t2	16731025	8048074	0.50326	4995800
SLX-21036_JQ1.pc10.B2.t3	19999300	10696261	0.476691	7264336
SLX-21390_JQ1.pc10.B2.t4	15822912	7539153	0.494015	4828069
SLX-21390_JQ1.pc10.B2.t5	17186236	8585979	0.510393	5203483
SLX21703_CnT_JQ1Bio_U2OS.PCR-10.B1.t1	18631767	7045395	0.298639	9317139
SLX21703_CnT_JQ1Bio_U2OS.PCR-10.B1.t2	15040683	6477508	0.253946	10504572
SLX21703_CnT_JQ1Bio_U2OS.PCR-10.B1.t3	17279804	7414478	0.281426	10578603
SLX21703_CnT_JQ1Bio_U2OS.PCR-10.B1.t4	18744052	7520802	0.278022	10897140
SLX21703_CnT_JQ1Bio_U2OS.PCR-10.B1.t5	15896700	5346263	0.236605	9450564
SLX21704_CnT_JQ1Bio_U2OS.PCR-10.B2.t1	10345020	6038621	0.262376	9401269
SLX21704_CnT_JQ1Bio_U2OS.PCR-10.B2.t2	11881450	5910171	0.267526	8983080
SLX21704_CnT_JQ1Bio_U2OS.PCR-10.B2.t3	11573883	5880726	0.280753	8413200
SLX21704_CnT_JQ1Bio_U2OS.PCR-10.B2.t4	9436401	4886790	0.282252	6944804
SLX21704_CnT_JQ1Bio_U2OS.PCR-10.B2.t5	7567919	3923580	0.240829	6786543

Supplementary Table 2: DNA oligomers used in the FRET melting assays

Oligomer name	Sequence (5' to 3')	Ref
**G4 Kit1**	FAM-**GGG**A**GGG**CGCT**GGG**AGGA**GGG**-TAMRA	[9]
**G4 Myc**	FAM-TGA**GGG**T**GGG**TA**GGG**T**GGG**TAA-TAMRA	[9]
**G4 Telo**	FAM-**GGG**TTA**GGG**TTA**GGG**TTA**GGG**-TAMRA	[9]
**dsDNA**	FAM-TATAGCTATA-HEG-TATAGCTATA-TAMRA	[9]

Supplementary Table 3: ΔT_m caused by small molecule ligands at 1 μM in FRET melting assays

ΔT_m	G4 c-Kit1	G4 Myc	G4 Telo	dsDNA
**PDS**	22 °C	9 °C	23 °C	0 °C
**PDS-btn**	20 °C	7 °C	18 °C	0 °C
**PhenDC3**	30 °C	10 °C	25 °C	1 °C
**PhenDC3-btn**	19 °C	7 °C	15 °C	0 °C

Reference

1. Müller, S., Kumari, S., Rodriguez, R. & Balasubramanian, S. Small-molecule-mediated G-quadruplex isolation from human cells. *Nat. Chem.* **2**, 1095-1098 (2010).
2. Lefebvre, J., Guetta, C., Poyer, F., Mahuteau-Betzer, F. & Teulade-Fichou, M.-P. Copper–Alkyne Complexation Responsible for the Nucleolar Localization of Quadruplex Nucleic Acid Drugs Labeled by Click Reactions. *Angew. Chem. Int. Ed.* **56**, 11365-11369 (2017).
3. Filippakopoulos, P. et al. Selective inhibition of BET bromodomains. *Nature* **468**, 1067-1073 (2010).
4. Zhang, X., Spiegel, J., Martínez Cuesta, S., Adhikari, S. & Balasubramanian, S. Chemical profiling of DNA G-quadruplex-interacting proteins in live cells. *Nat. Chem.* **13**, 626-633 (2021).
5. Tjandra, K.C. et al. Identification of Novel Medulloblastoma Cell-Targeting Peptides for Use in Selective Chemotherapy Drug Delivery. *J. Med. Chem.* **63**, 2181-2193 (2020).
6. Landt, S.G. et al. ChIP-seq guidelines and practices of the ENCODE and modENCODE consortia. *Genome Res.* **22**, 1813-1831 (2012).
7. Meers, M.P., Tenenbaum, D. & Henikoff, S. Peak calling by Sparse Enrichment Analysis for CUT&RUN chromatin profiling. *Epigenetics Chromatin* **12**, 42 (2019).
8. Kaya-Okur, H.S., Janssens, D.H., Henikoff, J.G., Ahmad, K. & Henikoff, S. Efficient low-cost chromatin profiling with CUT&Tag. *Nat. Protoc.* **15**, 3264-3283 (2020).
9. Le, D.D., Di Antonio, M., Chan, L.K.M. & Balasubramanian, S. G-quadruplex ligands exhibit differential G-tetrad selectivity. *ChemComm* **51**, 8048-8050 (2015).
